# Cell‐type‐specific role of lamin‐B1 in thymus development and its inflammation‐driven reduction in thymus aging

**DOI:** 10.1111/acel.12952

**Published:** 2019-04-09

**Authors:** Sibiao Yue, Xiaobin Zheng, Yixian Zheng

**Affiliations:** ^1^ Department of Embryology Carnegie Institution for Science Baltimore Maryland; ^2^ Department of Biology Johns Hopkins University Baltimore Maryland

**Keywords:** aging, inflammation, lamin-B1, senescence, thymus involution

## Abstract

Cellular architectural proteins often participate in organ development and maintenance. Although functional decay of some of these proteins during aging is known, the cell‐type‐specific developmental role and the cause and consequence of their subsequent decay remain to be established especially in mammals. By studying lamins, the nuclear structural proteins, we demonstrate that lamin‐B1 functions specifically in the thymic epithelial cells (TECs) for proper thymus organogenesis. An up‐regulation of proinflammatory cytokines in the intra‐thymic myeloid immune cells during aging accompanies a gradual reduction of lamin‐B1 in adult TECs. We show that these cytokines can cause senescence and lamin‐B1 reduction of the young adult TECs. Lamin‐B1 supports the expression of TEC genes that can help maintain the adult TEC subtypes we identified by single‐cell RNA‐sequencing, thymic architecture, and function. Thus, structural proteins involved in organ building and maintenance can undergo inflammation‐driven decay which can in turn contribute to age‐associated organ degeneration.

## 1 INTRODUCTION

Cell and tissue architectural proteins, such as extracellular matrices, cytoskeleton, and nucleoskeleton, are involved in maintaining cell and tissue shapes. It is thus generally accepted that these proteins are required for tissue/organ building, and their “wearing and tearing” during the process of maintaining tissue/organ functions could contribute toward aging. However, due to their often ubiquitous presence in all cell types, not much effort has been devoted to understand whether they have cell‐type‐specific roles in organ building, if organ aging is caused by the cell‐type‐specific decay of these proteins, and the cause of such decay during aging.

Among the structural proteins, lamins, the major component of the nuclear lamina that forms a filamentous meshwork in the nucleus has been implicated in proper organogenesis (Coffinier et al., 2011; Kim et al., 2011). Interestingly, reduction of lamin‐B1 is found in the aging human skins (Dreesen et al., 2013), Alzheimer's disease patient brains (Frost, Bardai, & Feany, 2016), and various *Drosophila* organs (Chen, Zheng, & Zheng, 2014; Tran, Chen, Zheng, & Zheng, 2016), but the cause of such reduction and its impact on organ function, especially in mammals, remain poorly understood.

Elevated proinflammatory cytokines in aging animals, including humans, have been shown to contribute to various organ dysfunctions and human diseases (Franceschi et al., 2000). Indeed, extensive studies in vitro have shown that proinflammatory cytokines can induce senescence of a number of tissue culture cells (Acosta et al., 2008; Dumont, Balbeur, Remacle, & Toussaint, 2000; Kuilman et al., 2008). For example, either overexpression of CXCR2 in human primary fibroblasts or treatment of these cells with IL‐1α or TNF‐α induces cellular senescence (Acosta et al., 2008; Dumont et al., 2000). These proinflammatory cytokines can also reinforce cellular senescence in other primary tissue culture cells triggered by forced oncogene expression (Kuilman et al., 2008). Despite these studies, however, the cell/tissue source of age‐associated inflammation and whether such inflammation disrupts structural proteins and thus contributes to organ aging remain unclear in any organism.

Considering the varied environments different tissues/organs reside in and the different functions they perform, it is highly likely that the inflammatory causes and consequences are different in different tissues and organisms. Cellular senescence triggered by inflammation has been implicated in aging and organ degeneration in mammal (Ren, Pan, Lu, Sun, & Han, 2009). The multitudes of senescence‐associated cellular changes have, however, made it difficult to pinpoint which of these changes makes a key contribution toward age‐associated organ dysfunction. Additionally, vertebrate organs often contain complex cell types, which makes it challenging to identify the cell source(s) and target(s) of inflammation that contribute to organ aging. Among many organs, the vertebrate thymus has a relatively simple stromal cell population called thymic epithelial cells (TECs) that are essential for thymic development, organization, and function (Anderson & Takahama, 2012). The TECs can thus serve as a relatively simple model to understand how inflammation and cellular senescence could influence structural proteins and in turn contribute to organ aging.

As a primary lymphoid organ, the thymus produces naïve T cells essential for adaptive immunity. Differentiated from the Foxn1‐positive progenitors, the TECs consist of cortical TECs (cTECs) and medullary TECs (mTECs) that make up the cortical and medullary compartments of the thymus, respectively (Boehm, Nehls, & Kyewski, 1995). Whereas the cTECs play a major role in the positive selection of T cells, the mTECs along with the thymic dendritic cells (DCs) mediate central tolerance by facilitating clonal deletion of self‐reactive T cells (Anderson & Takahama, 2012).

The age‐associated thymic involution or size reduction is known to contribute to the dysfunction of the immune system (Chinn, Blackburn, Manley, & Sempowski, 2012). Studies in mice have shown that thymic involution can be separated into two phases (Aw & Palmer, 2012; Aw, Silva, Maddick, von Zglinicki, & Palmer, 2008; Shanley, Aw, Manley, & Palmer, 2009). The first phase occurs within ~6 weeks after birth and is characterized by a rapid reduction of thymic size. This phase is referred to as the developmentally related involution and it does not negatively affect the immune system. The second phase of thymic involution occurs during the process of organism aging and is manifested as a gradual reduction of thymic size and naïve T‐cell production. Foxn1 reduction in TECs soon after birth appears to contribute to the first developmental phase of thymic involution (Chen, Xiao, & Manley, 2009; O'Neill et al., 2016; Rode et al., 2015), but the cause of the second age‐associated phase of involution is unknown.

We show that of the three lamins, only lamin‐B1 is required in TECs for the development and maintenance of the spatially segregated cortical and medulla compartments critical for proper thymic function. We identify several proinflammatory cytokines in aging thymus that trigger TEC senescence and TEC lamin‐B1 reduction. Importantly, we report the identification of 17 adult TEC subsets and show that lamin‐B1 reduction in postnatal TECs contributes to the age‐associated TEC composition change, thymic involution, reduced naïve T‐cell production, and lymphopenia.

## 2 RESULTS

### 2.1 Lamin‐B1 functions in TECs to support proper thymic development

**Figure 1:**
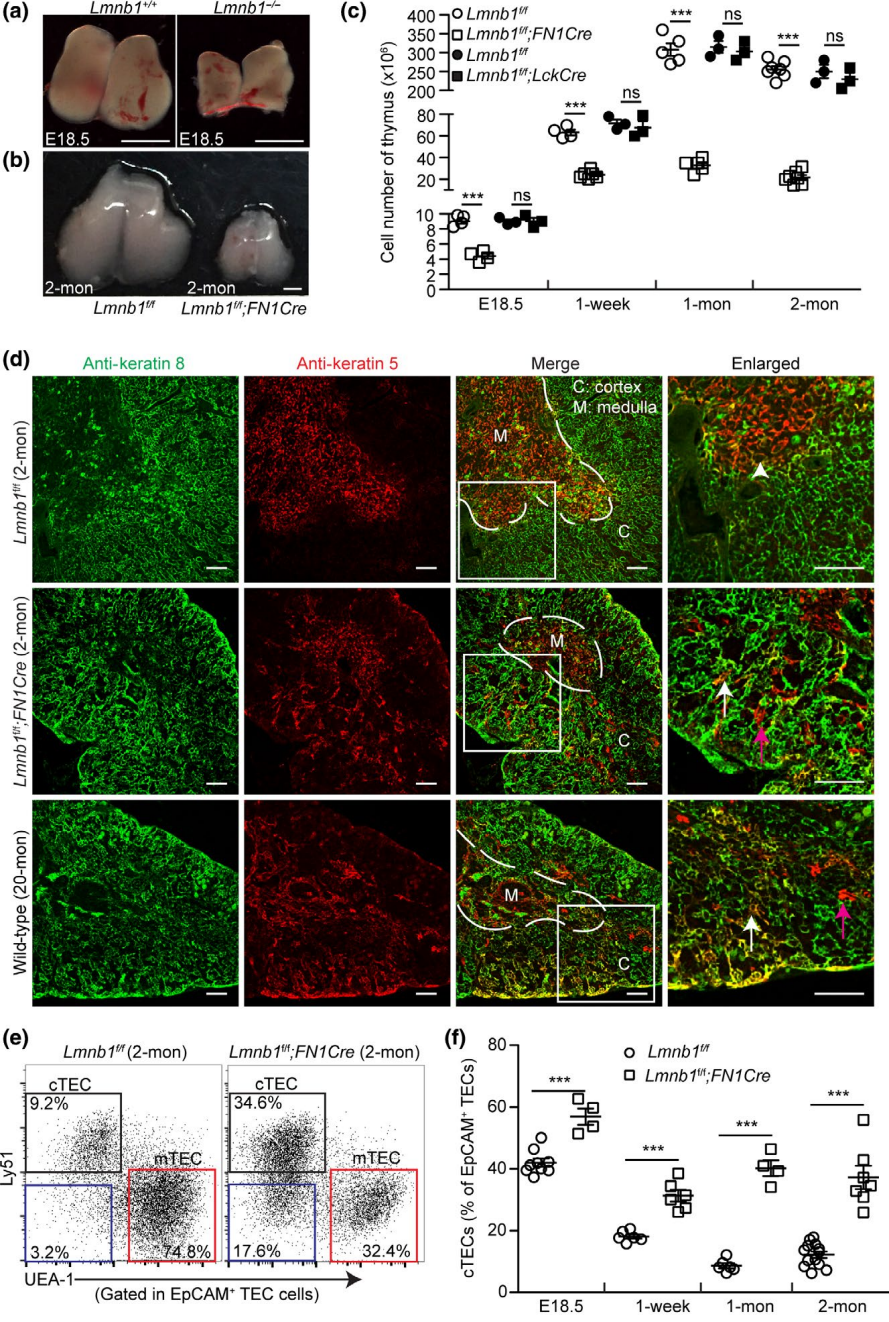
Effects of embryonic *Lmnb1* deletion in TECs on thymic organogenesis. (a,b) Representative images of thymuses from embryonic day 18.5 (E18.5) *Lmnb1^+/+^* and *Lmnb1^−/−^* mice (a) or 2‐mon‐old *Lmnb1^f/f^* and *Lmnb1^f/f^;Foxn1Cre* (*FN1Cre*) mice (b). Scale bars, 1mm. (c) Total thymic cell number counts from mice with the indicated ages and genotypes. (d) Distribution of cTECs (K8, green) and mTECs (K5, red) in thymuses with indicated genotypes as revealed by immunostaining. A 20‐mon‐old WT thymus is shown as an aged thymus control. White dash lines demarcate the cortical and medulla junction (CMJ) regions in the thymuses. A section of thymuses (white squares) in each genotype is enlarged to the right with the white arrowhead marking the K5^+^K8^+^ TECs in the CMJ in the 2‐mon‐old *Lmn1^f/f^* control thymus and the arrows marking the spreading of K5^+^K8^+^ TECs (white) and K5^+^ TECs (purple) into the cortical regions in the other thymuses. Scale bars, 100 μm. (e) Representative flow cytometric profiles showing the frequency of cTECs (Ly51^+^UEA‐1*^−^*, black squares) and mTECs (UEA‐1^+^Ly51*^−^*, red squares) from 2‐mon‐old *Lmnb1^f/f^* and *Lmnb1^f/f^;FN1Cre* thymuses. The displayed cells (each dot represents one cell) are gated first on CD45*^−^*EpCAM^+^ cells and then analyzed according to Ly51 and UEA‐1 to identify cTEC and mTEC subsets. The percentage of UEA‐1*^−^* Ly51*^−^* TECs is shown in the bottom left corner (blue squares). (f) Summary of the frequency of cTECs in *Lmnb1^f/f^* control and *Lmnb1^f/f^; FN1Cre* thymuses at the indicated ages. Each circle or square represents one control *Lmnb1^f/f^* and one *Lmnb1^f/f^;FN1Cre* mouse, respectively. Error bars, standard error of the mean (*SEM*) based on at least three independently analyzed mice. Student's *t* test: *p < 0.05, ** *p* < 0.01, ***p < 0.001; ns, not significant

To understand whether lamins play a role in thymus development, we first analyzed the thymuses in mice deleted of the lamin‐B1 gene, *Lmnb1*, in the germline and their littermate controls during embryogenesis. We found that the embryonic (E) day 18.5 (E18.5) *Lmnb1* null thymuses were smaller than the age‐matched littermate controls (Figure 1a). Since defects in thymocytes or TECs or both can result in small thymuses, we sought to identify the cell type where *Lmnb1*plays an important role during thymic organogenesis. We used the *Lmnb1^f/f^* allele derived from *Lmnb1* ^tm1a(EUCOMM)Wtsi^ from the EUCOMM project. These mice were crossed with the *Lck‐Cre* or *Foxn1‐Cre* (*FN1Cre*) mice to generate T‐cell‐ or TEC‐specific *Lmnb1* knockout mice, respectively (Gordon et al., 2007; Hennet, Hagen, Tabak, & Marth, 1995). We found that ablation of *Lmnb1* in TECs resulted in a significant reduction in the size and total cell number in the 2‐month (mon)‐old mouse thymuses compared to the littermate controls, whereas deleting *Lmnb1* in T cells had no apparent effect (Figure 1b,c). Since *FN1Cre* begins to express Cre at E11.5 in TECs (Gordon et al., 2007), we further analyzed thymuses in mice from E18.5 to 2 mon after birth. We found that the thymuses isolated from the *Lmnb1^f/f^;FN1Cre*mice were smaller in size and had reduced total cell numbers as compared to the littermate controls, whereas *Lmnb1*deletion in the *Lmnb1^f/f^;LckCre* mice had no such effect (Figure 1c). We also found that *Lmnb1*deletion in the TEC cells did not cause significant cell death in the thymus as compared to the littermate controls at all examined age stages (Supporting Information Figure S1a,b).

We next assessed the role of the other two lamins, lamin‐B2 encoded by *Lmnb2*and lamin‐A/C encoded by *Lmna,*in TECs. The conditional *Lmnb2* allele, *Lmnb2^f/f^*, was derived from *Lmnb2* ^tm1a(KOMP)Wtsi^ (KOMP project) by breeding with ACTB‐FLPe mice, which express FLP1 recombinase in a wide variety of cells including germ cells, to remove the neomycin cassette flanked by Frt sites. The *Lmna^f/f^* we generated in house was reported previously (Kim & Zheng, 2013). The *Lmnb2^f/f^*or *Lmna^f/f^* mice were crossed with *FN1Cre*mice. Genotyping and qPCR analyses revealed a similar deletion efficiency of all three lamin genes by *FN1Cre* in both cTECs and mTECs (Supporting Information Figure [Link], [Link], [Link], [Link]c,d). We found that depletion of either *Lmnb2* or *Lmna* in TECs had no obvious effect on thymic size and total cellularity (Supporting Information Figure [Link], [Link], [Link], [Link]e–g). These analyses show that lamin‐B1, but not lamin‐B2 and lamin‐A/C, in TECs supports the proper building of thymus during development.

### 2.2 Lamin‐B1 supports proper development of cortical and medulla thymic compartments

To analyze whether lamin‐B1 deletion affects the organization of TECs, we immunostained cTECs and mTECs using anti‐keratin 8 (K8) or 5 (K5) antibodies, respectively, in the thymuses from the 2‐mon‐old *Lmnb1^f/f^;FN1Cre*mice or their littermate controls. We found that lamin‐B1 deletion in TECs resulted in an intermingling of the cortical and medullary TEC compartments compared to the controls (Figure 1d). Interestingly, this intermingling of the two TEC compartments is highly reminiscent of the mixed cTEC and mTEC compartments known to occur in the old wild‐type (WT) mouse thymuses (Figure 1d). The breakdown of the cortical and medullary compartments is reflected in an increased presence of K5 and K8 double‐positive (K5^+^K8^+^) TECs throughout thymuses and the presence of K5^+^ mTECs outside of the medullary region of both the 2‐mon‐old *Lmnb1^f/f^;FN1Cre*and the 20‐mon‐old WT mouse thymuses, whereas in the young WT thymuses the K5^+^K8^+^ TECs were located in the cortical and medullary junction (CMJ) regions (Figure 1d).

The TECs that are EpCAM^+^can be further separated into mTECs and cTECs by flow cytometry as each subset of cells is either UEA‐1^+^ or Ly51^+^, respectively. Using flow cytometry, we further analyzed the mTECs (EpCAM^+^UEA‐1^+^Ly51*^−^*) and cTECs (EpCAM^+^UEA‐1*^−^*Ly51^+^) and found a pronounced reduction in the frequency of mTECs and a skewing of the TEC population toward cTECs in the *Lmnb1^f/f^;FN1Cre*thymuses compared to the littermate controls (Figure 1e,f). There was also a marked increase of a TEC subpopulation that did not express the canonical surface UEA‐1 or Ly51 (Figure 1e, blue squares) in the *Lmnb1^f/f^;FN1Cre*thymuses. These analyses demonstrate that lamin‐B1 is not required for the TEC lineage commitment but it plays a role in efficient TEC differentiation and TEC compartment organization during thymus organogenesis.

### 2.3 Embryonic lamin‐B1 deficiency in TECs impairs positive selection of conventional αβT cells

**Figure 2:**
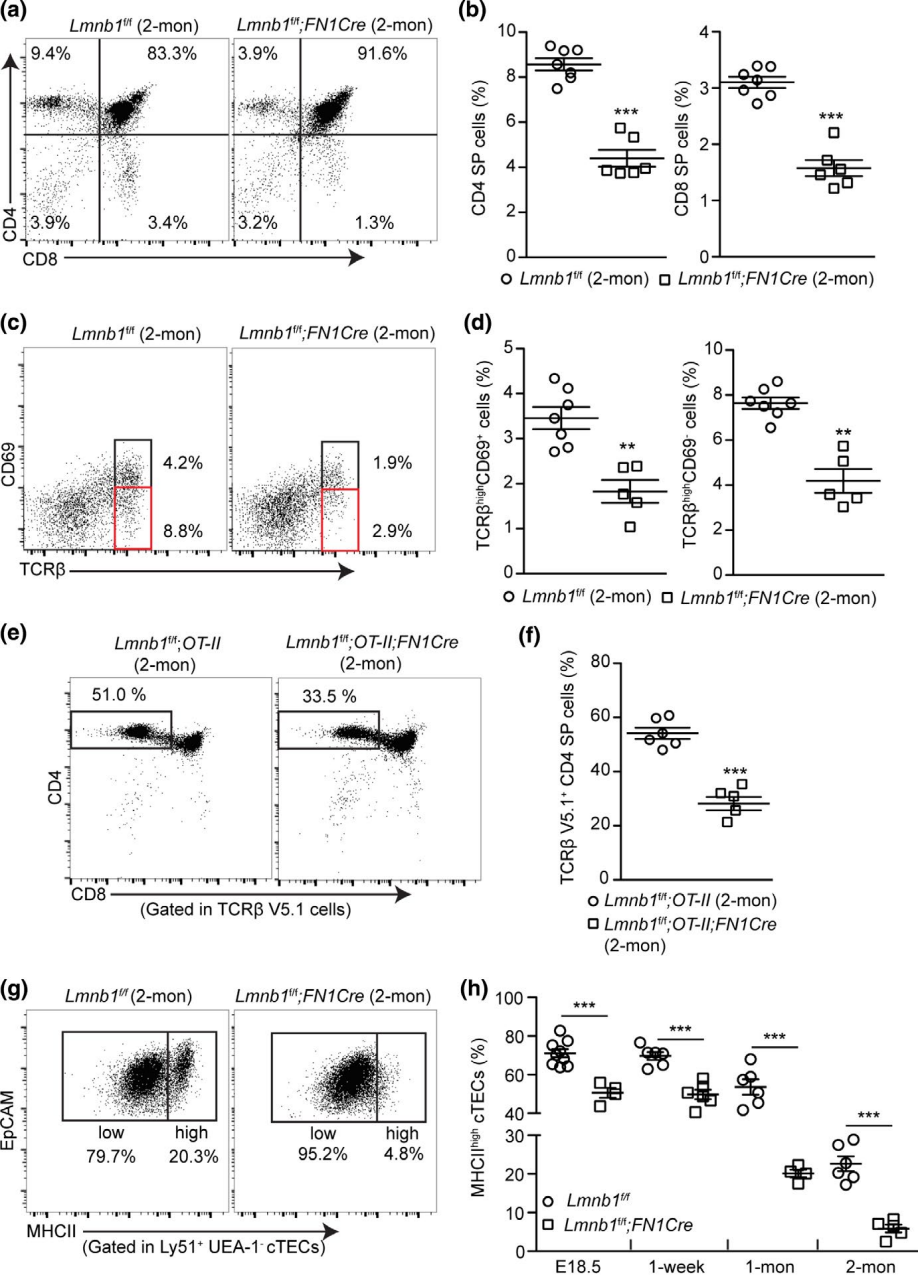
Effects of developmental TEC *Lmnb1* deletion on the positive selection of αβT cells in mouse thymus. (a) Representative flow cytometry profiles showing a reduction of CD4^+^ single‐positive (CD4^+^ SP) and CD8^+^ SP thymocytes in the 2‐mon‐old *Lmnb1^f/f^;FN1Cre* mouse thymuses compared to that of the *Lmnb1^f/f^* controls. (b) Quantifications of the frequency of CD4^+^ and CD8^+^ SP thymocytes within the gated live cells in the 2‐mon‐old *Lmnb1^f/f^* control (*n* = 7) and *Lmnb1^f/f^;FN1Cre* (*n* = 6) mouse thymuses. (c) Flow cytometry analysis of TCRβ and CD69 expression on thymocyte subsets undergoing positive selection in 2‐mon‐old *Lmnb1^f/f^* control and *Lmnb1^f/f^;FN1Cre* mouse thymuses. Black and red boxes show the TCRβ^high^CD69^+^ or TCRβ^high^CD69*^−^* thymocytes. (d) Quantification of the frequency of the TCRβ^high^CD69^+^ and TCRβ^high^CD69*^−^* cells in the 2‐mon‐old *Lmnb1^f/f^* control (*n* = 7) and *Lmnb1^f/f^;FN1Cre* (*n* = 5) mouse thymuses. (e) Flow cytometry analysis showing a reduction of CD4^+^ SP thymocytes in the 2‐mon‐old *Lmnb1^f/f^;OT‐II;F1NCre* thymuses (*n* = 5) compared to the *Lmnb1^f/f^;OT‐II* thymuses (*n* = 6). (f) Quantification of the frequency of CD4^+^ SP thymocytes within the gated OT‐II transgenic TCR (TCRβ V5.1) thymocytes in experiments shown in (e). (g) Representative flow cytometry plots of staining for MHCII^high^ and MHCII^low^ cTECs from 2‐mon‐old *Lmnb1^f/f^* and *Lmnb1^f/f^;FN1Cre* thymuses. (h) A summary showing the reduction of the most differentiated MHCII^high^ cTECs in the *Lmnb1^f/f^;FN1Cre* thymuses compared to the *Lmnb1^f/f^* control thymuses at the indicated ages. Each circle or square represents one *Lmnb1^f/f^* control or *Lmnb1^f/f^;FN1Cre* mouse, respectively. Error bars, *SEM* from at least four independently analyzed mice. Student's *t* test: *p < 0.05, **p < 0.01, ***p < 0.001; ns, not significant

Since the disrupted mTEC and cTEC compartmentalization and composition upon lamin‐B1 deletion could affect proper TEC niche formation, thereby affecting T‐cell development (thymopoiesis) (Anderson & Takahama, 2012), we examined the impact of lamin‐B1 deficiency in TECs on thymopoiesis. We first analyzed the CD4 or CD8 single‐positive (SP) thymocytes and found a 50%–60% reduction in each of these thymocytes in the 2‐mon‐old *Lmnb1^f/f^;FN1Cre*thymuses compared to the littermate controls (Figure 2a,b). Flow cytometry analyses of cell surface expression of TCRβ chain and CD69 on thymocytes further revealed an ~50% reduction of the TCRβ^high^CD69^+^and mature TCRβ^high^CD69*^−^* populations in the 2‐mon‐old *Lmnb1^f/f^;FN1Cre* mice (Figure 2c,d). We further confirmed this finding by using the OT‐II transgenic TCR mouse model and found that depletion of *Lmnb1* by *FN1Cre* led to a substantial reduction of the transgenic OT‐II CD4^+^SP thymocytes (Figure 2e,f).

We next studied how lamin‐B1 deficiency in TECs can affect TEC maturation as inappropriate TEC development would affect αβT‐cell production. TECs can be separated into the MHCII^low^ immature and the MHCII^high^ mature and more differentiated TECs. A decline in the number of mature MHCII^high^TECs is a hallmark of the age‐associated thymus change in mice (Chinn et al., 2012). Strikingly, we found an ~5‐fold reduction of the mature MHCII^high^ cTECs, in the 2‐mon‐old *Lmnb1^f/f^;FN1Cre*mice compared to littermate controls (Figure 2g,h). A reduction, albeit less pronounced, in the frequency of the MHCII^high^ mature mTECs was also observed in the 2‐mon‐old *Lmnb1^f/f^;FN1Cre*mice (*Lmnb1^f/f^*: 54% ± 4%; *Lmnb1^f/f^;FN1Cre:*42% ± 3%; *p* value = 0.003). Thus, lamin‐B1 plays a critical role in TECs, particularly in cTECs, to promote efficient TEC differentiation and maturation from their progenitors, thereby supporting the organization of thymic cortical and medulla compartments and αβT‐cell generation.

Interestingly, the frequencies of the double‐negative (DN) thymocytes and the γδT cells, whose development is not dependent on the expression of MHCII in cTECs (Chien, Meyer, & Bonneville, 2014), are similar in the 2‐mon‐old *Lmnb1^f/f^;FN1Cre*and the littermate control thymuses (Supporting Information Figure [Link], [Link], [Link], [Link]). These results suggest that lamin‐B1 is dispensable in cTECs to support the early stages of T‐cell development.

### 2.4 Gradual lamin‐B1 reduction in TECs during age‐associated thymic involution

**Figure 3:**
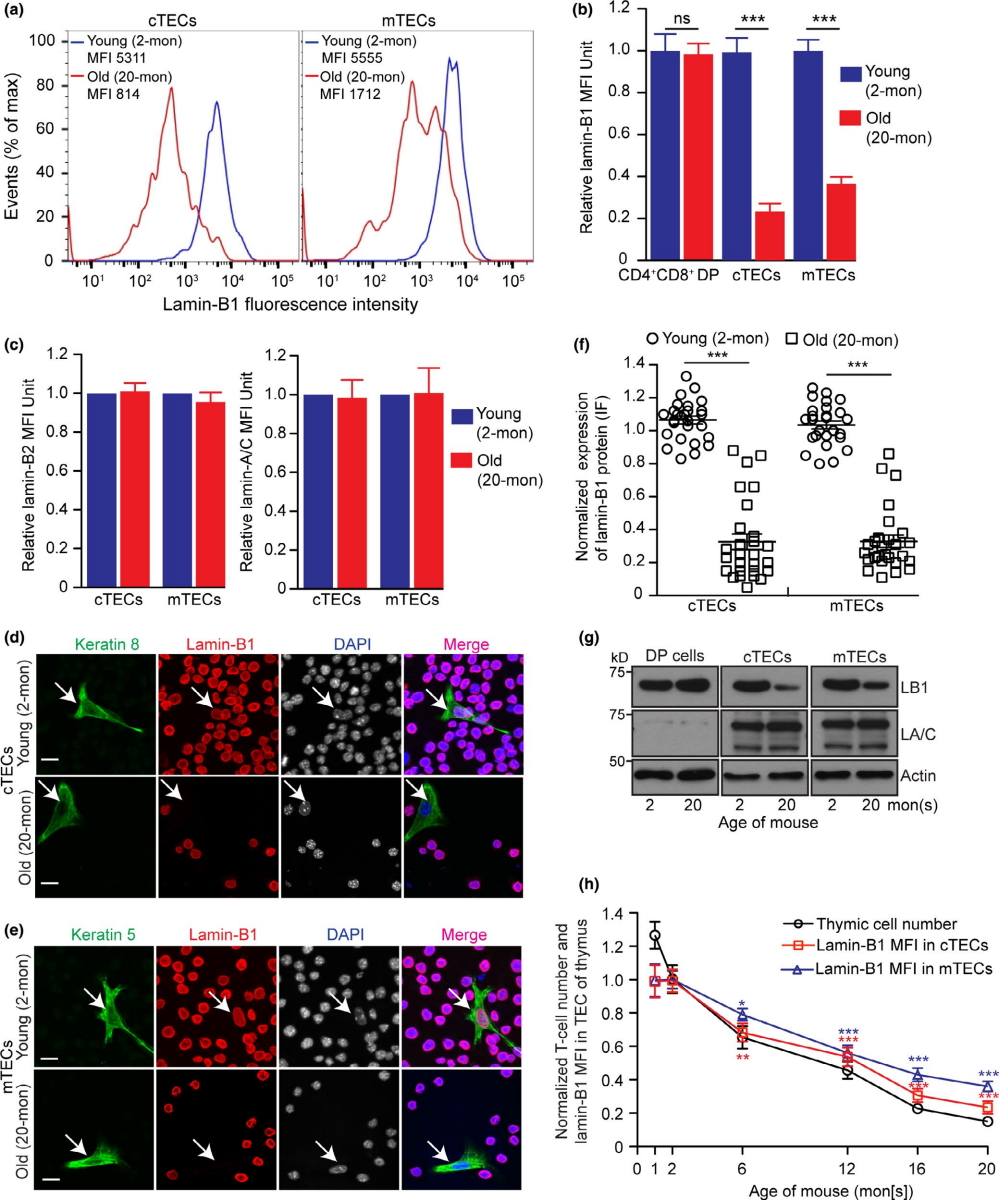
Effects of aging on lamin‐B1 protein levels in TECs. (a) Flow cytometry analyses of lamin‐B1 levels in cTECs (left) and mTECs (right) from 2‐ (young, blue) or 20‐mon‐old (old, red) wild‐type mouse thymuses. Shown are representative histogram plots (out of >3 independent experiments) with the mean fluorescence intensities (MFI) of lamin‐B1 indicated. (b) Quantification of lamin‐B1 MFI in the CD4^+^CD8^+^ double‐positive (DP) thymocytes, cTECs, and mTECs in the young and old WT mouse thymuses. (c) Quantification of lamin‐B2 (left) and lamin‐A/C (right) MFI in the cTECs and mTECs in the young and old WT mouse thymuses from two mice analyzed on different days. (d,e) Immunostaining of lamin‐B1 (red) in cTECs (d) and mTECs (e) along with the spiked‐in RAW264.7 cells (ATCC, #TIB‐71) as an internal control for lamin‐B1 staining intensity. Keratin 8 or 5 (green) labels cTECs (d) or mTECs (e) isolated from 2‐ or 20‐mon WT mouse thymuses, respectively. (f) Quantification of lamin‐B1 fluorescence intensity in cTECs or mTECs from (d) and (e). The average lamin‐B1 fluorescence intensity in TECs and the surrounding spiked‐in RAW264.7 cells were measured, and the TEC lamin‐B1 fluorescence intensities were plotted relative to the lamin‐B1 intensity in spiked‐in RAW264.7 cells which was set to 1. 25 cTECs or mTECs were measured in three independent experiments. (g) Western blotting analyses of lamin‐B1 and lamin‐A/C in CD4^+^CD8^+^ DP cells, cTECs, or mTECs in 2‐ or 20‐mon thymuses. One 2‐mon‐old or five pooled 20‐mon‐old thymuses were used for each Western blotting analysis. β‐actin is used for loading control. Shown is one representative Western blot of two independent experiments. (h) Quantification of the total thymic cell numbers (black) and lamin‐B1 MFI in cTECs (red) or mTECs (blue) at the indicated ages. Total thymic cell number or MFI of lamin‐B1 in cTECs or mTECs was plotted relative to those of 2‐mon WT thymus, which was defined as 1 (≥three independent experiments). Error bars, *SEM*. Scale bars, 20 μm. Student's *t* test: *p < 0.05, **p < 0.01, ***p < 0.001, ns: not significant

Since the disruption of TEC compartments and T‐cell development in the 2‐mon‐old *Lmnb1^f/f^;FN1Cre*thymuses resembled the changes in the old mouse thymus, we asked whether lamin‐B1 reduction may accompany the age‐associated thymic involution. The mouse thymus first undergoes a rapid phase of involution in the first several weeks after birth followed by a second age‐associated phase of gradual involution. We immunostained lamin‐B1, ‐B2, and ‐A/C, in the CD4^+^CD8^+^double‐positive (DP) thymocytes, cTECs, and mTECs isolated from 2‐ and 20‐mon‐old WT mouse thymuses and analyzed the protein levels using flow cytometry. Quantification of the mean fluorescence intensity (MFI) revealed that lamin‐B1 was reduced by >65% in cTECs and mTECs, but not in DP thymocytes, in old WT mice compared to young WT mice (Figure 3a,b). The lamin‐B2 and lamin‐A/C levels remained the same upon aging in these cells (Figure 3c). Consistent with early studies (Aw & Palmer, 2012; Aw et al., 2008; Shanley et al., 2009), we did not observe significant changes of total cell death in the WT mouse thymus at all examined ages, indicating that lamin‐B1 loss in TEC upon aging does not contribute to cell death in thymus (Supporting Information Figure [Link], [Link], [Link], [Link]a).

To further confirm lamin‐B1 reduction upon aging in TECs, we used fluorescence‐activated cell sorting (FACS) to isolate cTECs and mTECs. We mixed an adherent keratin‐negative cell line (RAW264.7 from ATCC #TIB‐71) with the sorted cTECs or mTECs to control for variability of immunostaining (Figure 3d,e). By quantifying lamin‐B1 intensity in individual TECs and normalizing against the lamin‐B1 level of the spike‐in RAW264.7 cells, we found that ~80% cTECs or mTECs exhibited >50% lamin‐B1 reduction in the old thymus (Figure 3f). Western blotting analyses of the isolated TECs also showed a large reduction of lamin‐B1 level upon aging, whereas lamin‐A/C remained unchanged (Figure 3g). Interestingly, we found that lamin‐B1 reduction in TEC upon aging did not cause obvious alteration of nuclear morphology based on DAPI staining (Figure 3d,e; Supporting Information Figure [Link], [Link], [Link], [Link]b,c). Since we have shown that it is the total lamin concentration that is important in maintaining nuclear structure (Guo, Kim, Shimi, Goldman, & Zheng, 2014), the reduction (not complete loss) of only lamin‐B1 may not decrease the total lamin level in the TEC nucleus sufficiently to alter nuclear morphology.

We next analyzed how the kinetics of lamin‐B1 reduction in TECs correlated with the two phases of thymic involution. We quantified the total thymic cell number as an indicator for thymic involution and used FACS to determine lamin‐B1 level by MFI in all TECs. We found that mouse thymuses exhibited ~20%–25% reduction of total cell numbers within the first two 2 months after birth which correlated with the first phase of thymic involution, but lamin‐B1 in TECs remained unchanged in this time window. By contrast, a gradual reduction of lamin‐B1 in TECs occurred and it coincided with the second phase of gradual thymic aging as judged by total thymic cellularity (Figure 3h). Thus, lamin‐B1 reduction correlates with the age‐associated thymic involution.

### 2.5 Lamin‐B1 reduction in TECs accompanies age‐associated thymic inflammation and can be triggered by proinflammatory cytokines

**Figure 4:**
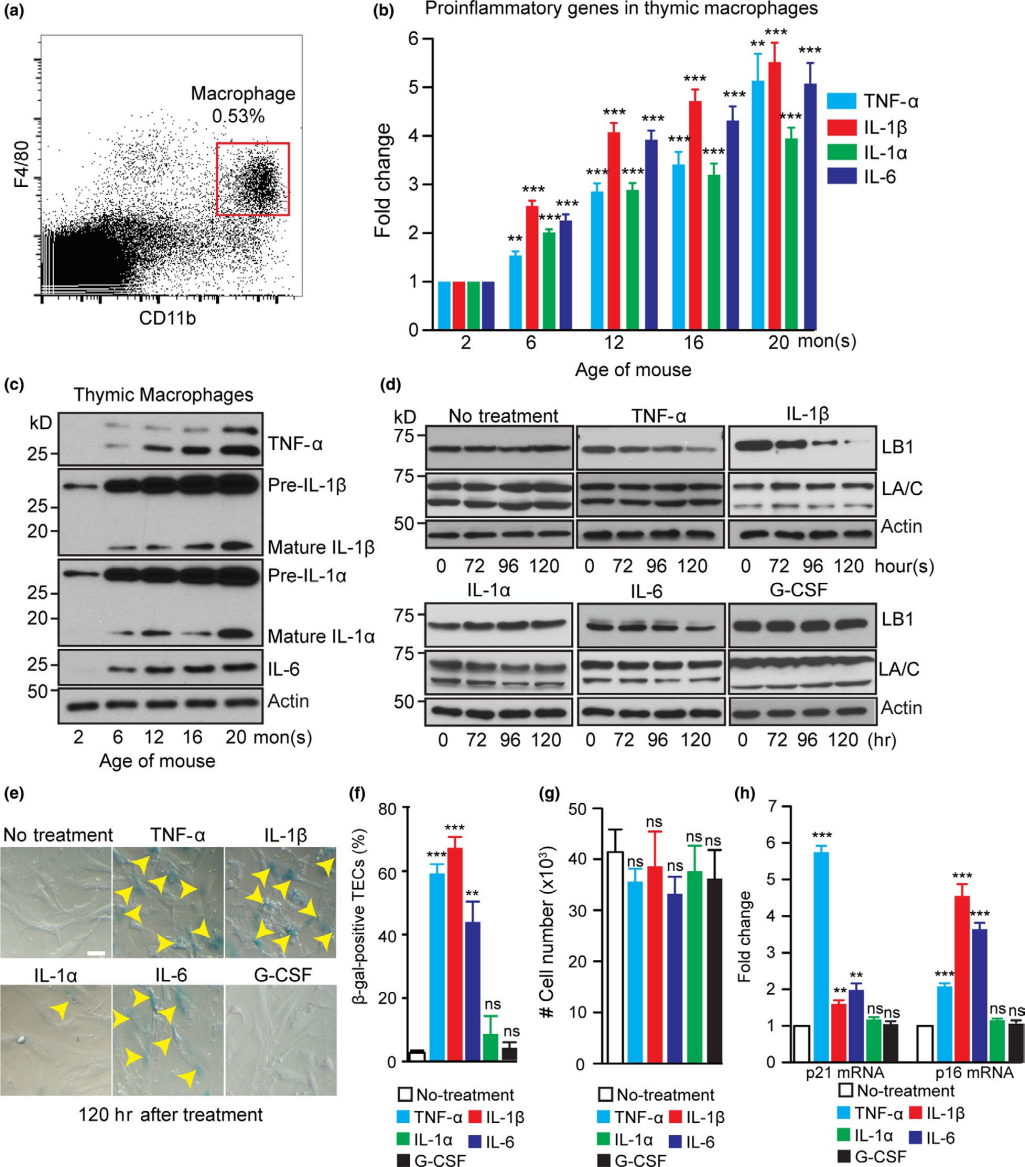
Effects of inflammatory cytokines on the TEC lamin‐B1 level. (a) The flow cytometry gating strategy for sorting macrophages from a 2‐mon‐old WT thymus. Macrophages were identified based on the expression of F4/80 and CD11b. (b) RT–qPCR analyses of TNF‐α, IL‐1β, IL‐1α, and IL‐6 in macrophages isolated from WT thymuses at the indicated ages. The increased expression was plotted relative to the 2‐mon‐old thymus, which was set to 1. (c) A representative Western blotting analysis of TNF‐α, IL‐1β, IL‐1α, and IL‐6 in macrophages isolated from WT thymuses at the indicated ages. β‐actin, loading control. (d) Western blotting analyses of lamin‐B1 and lamin‐A/C in cultured primary TECs treated with TNF‐α (10 ng/ml), IL‐1β (20 ng/ml), IL‐1α (20 ng/ml), IL‐6 (20 ng/ml), and G‐CSF (20 ng/ml) for the indicated hours. (e) Increased TEC senescence as judged by β‐galactosidase staining (yellow arrowheads) upon treatment by TNF‐α, IL‐1β, and IL‐6, but not by IL‐1α and G‐CSF. (f) Quantification of the percentage of β‐galactosidase‐positive primary TECs. Total 150 cells from 3 biological replicates were counted for each experiment. (g) The cell number counts from in vitro cultured primary TECs after treating with indicated inflammatory cytokines for 120 hr. 5 x10^4^ freshly isolated mature TECs were plated in each condition. (h) RT–qPCR analyses showing the up‐regulation of p21 and p16 in the primary TECs upon treatment with the indicated proinflammatory cytokines. Scale bar, 20 μm. Error bars, *SEM* from three biological repeats. Student's *t* test: *p < 0.05, **p < 0.01, ***p < 0.001, ns: not significant

Age‐associated increase in intra‐thymic lipotoxic danger signals has been shown to lead to caspase‐1 activation via the Nlrp3 inflammasomes in thymic macrophages, which can in turn promote thymic inflammation and age‐related thymic demise (Youm et al., 2012), but the cellular and molecular targets of this inflammation in thymus are unknown. We first examined whether the elevated inflammatory state could correlate with age‐related thymic involution by applying FACS to isolate macrophages in thymuses dissected from 2‐, 6‐, 12‐, 16‐, and 20‐mon‐old mice (Figure 4a). Reverse transcriptase–quantitative polymerase chain reaction (RT–qPCR) and Western blotting analyses were used to measure the key proinflammatory cytokines, TNF‐α, IL‐1β, IL‐1α, and IL‐6, that have been implicated in thymic degeneration (Billard, Gruver, & Sempowski, 2011). We found that the thymic macrophages exhibited a significantly increased expression of proinflammatory cytokines by 6 months of age and the levels continued to increase with aging (Figure 4b,c). Similar to the thymic macrophages, we found that the thymic Sirpα^+^dendritic cells (DCs) (Ki et al., 2014) also exhibited an increased expression of these proinflammatory cytokines (Supporting Information Figure [Link], [Link], [Link], [Link]a–d).

Since by 6 months of age, the lamin‐B1 level in TECs was reduced by ~30% (see Figure 3h), we reasoned that the increased proinflammatory cytokines detected in thymus at this age could contribute to lamin‐B1 reduction in TECs. To test this, we isolated and cultured primary TECs from 2‐mon‐old mouse thymuses and treated these cells with TNF‐α, IL‐1β, IL‐1α, and IL‐6. Since G‐CSF is among the top genes exhibiting a strong increase in expression during intra‐thymic inflammatory response in the endotoxin‐induced acute thymic involution (Billard et al., 2011), we also included G‐CSF in our in vitro treatment. We found that in vitro culturing of the freshly isolated primary mature TECs (see method for TEC isolation) without any treatment resulted in a gradual reduction of the number of cells within 6 days, which was most likely caused by the ~4%–6% of TECs apoptosis (Supporting Information Figure [Link], [Link], [Link], [Link]e). Therefore, we performed the inflammatory cytokine treatment for 5 days. We found whereas TNF‐α, IL‐1β, and IL‐6 induced a gradual reduction of lamin‐B1 without affecting lamin‐A/C, IL‐1α and G‐CSF had no effect on either lamin in the mature TECs (Figure 4d). This shows that TEC lamin‐B1 levels are sensitive to certain proinflammatory cytokines. Since proinflammatory cytokines are known to trigger various signaling pathways to initiate and maintain cellular senescence (Acosta et al., 2008; Kuilman et al., 2008) and since the senescence of human and mouse fibroblasts is accompanied by lamin‐B1 reduction (Coppe et al., 2008; Dreesen et al., 2013), we examined whether TNF‐α, IL‐1β, and IL‐6 triggered TEC senescence. By quantifying cells positive for β‐galactosidase, a senescence marker, we found that > 40% of TECs underwent senescence after treatment by TNF‐α, IL‐1β, and IL‐6, but not by IL‐1α and G‐CSF (Figure 4e,f). We found that by 120 hr of treatment with different cytokines, similar numbers of cells remained in each culture compared to no treatment controls (Figure 4g). RT–qPCR analyses of p21 and p16, two cell cycle inhibitors involved in p53‐ and retinoblastoma protein (Rb)‐mediated senescence, respectively (Rodier & Campisi, 2011), showed that TNF‐α induced a strong up‐regulation of p21 expression while IL‐1β and IL‐6 mainly engaged in inducing p16 up‐regulation (Figure 4h). These findings suggest that the age‐associated increase of thymic TNF‐α, IL‐1β, and IL‐6 can trigger lamin‐B1 reduction in TECs at least in part by inducing cellular senescence.

Thymic involution also occurs under pathophysiological conditions, including chronic and acute infection (Chinn et al., 2012). We tested whether lamin‐B1 reduction in TECs could be stimulated by cytokines via injecting lipopolysaccharide (LPS), which is known to cause endotoxemia and acute thymic atrophy (Billard et al., 2011). Consistent with the previous report, intraperitoneal injection (IP) of one dose of LPS (100 μg) (Supporting Information Figure [Link], [Link], [Link], [Link]f) induced acute thymic involution within 48 hr as measured by total thymic cell number counts (Supporting Information Figure [Link], [Link], [Link], [Link]g). By 48 hr post‐LPS injection, we found a significant reduction of lamin‐B1 levels in FACS‐sorted mTECs by Western blotting analyses, while lamin‐A/C did not exhibit obvious changes (Supporting Information Figure [Link], [Link], [Link], [Link]h). Due to a very small number of cTECs present in the LPS‐treated mouse thymuses, we were unable to assess lamin protein levels in these cells. Taken together, these analyses suggest that age‐associated increase in the proinflammatory cytokines TNF‐α, L‐1β, and IL‐6 can induce TEC senescence and lamin‐B1 reduction.

### 2.6 Lamin‐B1 is required in TECs to support adult thymus organization in part by maintaining proper gene expression

**Figure 5:**
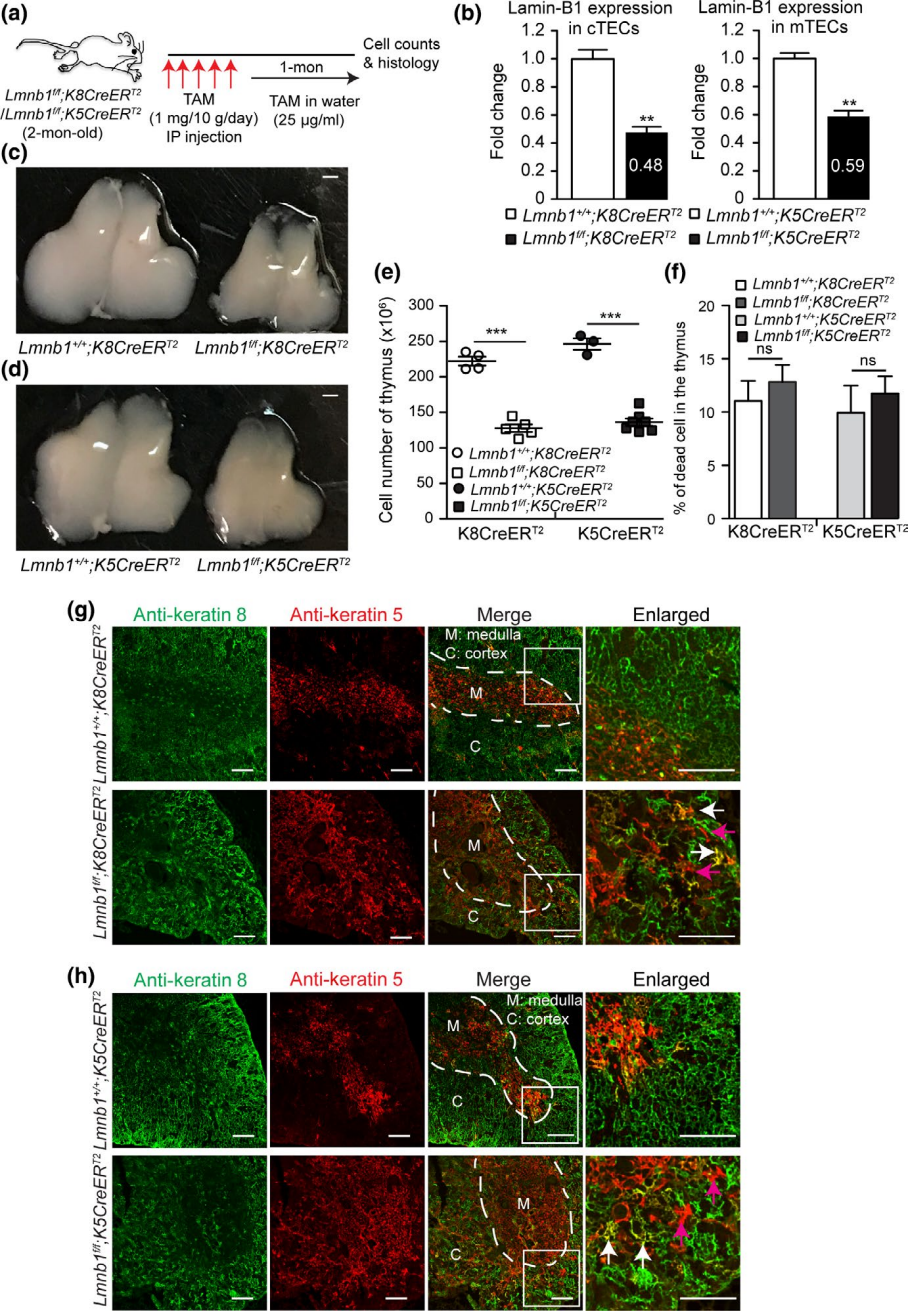
Effects of postnatal lamin‐B1 deficiency in TECs on thymus size and organization. (a) The tamoxifen (TAM)‐mediated *Lmnb1* deletion scheme. Keratin 8 (*K8*)‐ or keratin 5 (*K5*)‐driven *CreER^T2^* was used to delete *Lmnb1* in cTECs or mTECs, respectively, by injecting TAM followed by feeding in TAM‐containing drinking water before analyses. (b) RT–qPCR analyses of lamin‐B1 mRNA levels revealing the *Lmnb1*deletion efficiency by *K8CreER^T2^* in cTECs (left) or by *K5CreER^T2^* in mTECs (right). The average mRNA levels of lamin‐B1 were calculated from 3 independent experiments with the control set to 1. (c,d) Representative images of thymuses from mice with indicated genotypes after TAM treatment as shown in (a). Scale bars, 1 mm. (e) The total thymic cells were counted and plotted for indicated genotypes as shown. (f) Summary of cell death in the thymus from mice with the indicated ages and genotypes. The dead cells were identified as DAPI^+^ populations by flow cytometry. (g,h) Keratin staining of thymuses from mice with indicated genotypes treated by TAM as shown in (a). White dash lines demarcate the cortical and medulla junction (CMJ) regions in the thymus. A section (white squares) of the thymus in each genotype was enlarged to show the well‐defined (in the controls) or disorganized (in the mutants) CMJ. Arrows mark the spreading of K5^+^K8^+^ TECs (white) and K5^+^ TECs (purple) into the cortical regions in the in *Lmn1^f/f^;K8CreER^T2^* (g) or *Lmn1^f/f^;K5CreER^T2^* (h) thymuses. Scale bars, 100 μm. Error bars, *SEM* from ≥ 3 independent experiments. Student's *t* test: *p < 0.05, **p < 0.01, ***p < 0.001, ns: not significant

The role of TEC lamin‐B1 in thymus development prompted us to ask whether during adulthood lamin‐B1 is required for maintaining thymus structure and function, and if the age‐associated lamin‐B1 reduction in TECs contributes to thymic involution. We employed *K8CreER^T2^* or *K5CreER^T2^* to delete *Lmnb1* in either cTECs or mTECs, respectively, in the adult thymus by injecting tamoxifen (TAM) starting at 2 months of age (Figure 5a) (Cheng et al., 2010). It is important to note that cTEC‐ and mTEC‐restricted lineages are initially derived from bipotent TEC progenitors that may express both K5 and K8 (Klug et al., 1998). Thus, *K5CreER^T2^*may delete *Lmnb1* alleles in some cTEC sub‐lineages, whereas *K8CreER^T2^* may delete *Lmnb1* alleles in some mTEC sub‐lineages. Since TAM‐induced genomic excision has variable efficiency depending on the TAM dosages, *CreER^T2^* lines, and specific tissues, we tested different TAM doses. We found that 1mg TAM/10g body weight/day for five consecutive days followed by feeding the mice drinking water containing TAM (25µg/ml) resulted in an obvious reduction in thymic sizes in the *Lmnb1^f/f^;K8CreER^T2^* or *Lmnb1* ^f/f^ *;K5CreER^T2^* mice compared to the littermate controls and this procedure did not cause pronounced toxicity in all the injected mice. qRT–PCR revealed that this TAM regimen resulted in ~52% or 41% reduction of *Lmnb1* mRNA in the FACS‐sorted cTECs or mTECs, respectively, at one month after the last TAM injection (Figure 5b). The size and total cell number of thymuses in *Lmnb1^f/f^;K8CreER^T2^* or *Lmnb1* ^f/f^ *;K5CreER^T2^* mice were also reduced to 50%–60% of the littermate controls (Figure 5c–e). We found that *Lmnb1* deletion in adult TECs did not cause significant cell death in thymus as compared to the littermate controls (Figure 5f). These findings demonstrate that lamin‐B1 reduction in either cTECs or mTECs in the adult thymus can cause thymic involution.

We next performed K5 and K8 immunostaining to determine whether lamin‐B1 reduction affected the structural organization of the thymus. Compared to the littermate controls, the cortical and medullary compartments were disrupted with the spreading of K5^+^ mTECs into the cortical regions in the thymuses of TAM‐treated *Lmnb1^f/f^;K8CreER^T2^* (Figure 5g) and *Lmnb1^f/f^;K5CreER^T2^* mice (Figure 5h). Our data demonstrate that lamin‐B1 is required in the adult cTECs and mTECs to maintain the proper segregation of the cortical and medullary thymus architecture. Thus, the age‐associated lamin‐B1 reduction in TECs can contribute to the age‐associated phase of thymic involution.

To explore further how lamin‐B1 reduction contributes to thymic involution, we performed RNA‐seq of the control and lamin‐B1‐depleted TECs. Since the deletion efficiency of *Lmnb1* in mTECs by *K5CreER^T2^*is low and since the total mTEC number is also low in the TAM‐treated *Lmnb1^f/f^;K5CreER^T2^*thymus, a high percentage of our isolated mTECs were WT for *Lmnb1* which makes it difficult to interpret the RNA‐seq results. Therefore, we focused our analyses on the effect of lamin‐B1 deletion in cTECs. We considered the differential expression of genes as those with fold changes ≥2 than the control cTECs with the adjusted false discovery rate (FDR) <0.05 and *p* values <0.05 (see the method section). We found lamin‐B1 reduction in cTECs resulted in the transcriptional up‐ and down‐regulation of 533 and 778 genes, respectively (Supporting Information Figure [Link], [Link], [Link], [Link]a; Table S1 for the full list). DAVID Gene Ontology (GO) analysis revealed that lamin‐B1 deletion resulted in a dysregulation of genes implicated in cell adhesion, immune system process and development, T‐cell differentiation, and cytokine production that are relevant to thymus function (Supporting Information Figure [Link], [Link], [Link], [Link]b–d; Table S2 for the full list). Thus, lamin‐B1 plays an important role in the expression of genes required for maintaining TEC structure and niche, which would in turn support T‐cell development.

### 2.7 Lamin‐B1 is required for maintaining the distribution of adult TEC cell subsets in thymus

To understand further how natural aging and postnatal lamin‐B1 reduction contribute to TEC maintenance, we performed droplet‐based single cell RNA‐sequencing (scRNA‐seq) on isolated total TECs (DAPI*^−^* CD45*^−^* EpCAM^+^) from the WT young (2‐mon), WT old (20‐mon), and TAM‐treated 3‐mon‐old *Lmnb1^f/f^;K8CreER^T2^*mouse thymuses (see Figure 5a for the TAM scheme). For the reasons mentioned above in transcriptome analyses of TEC population, we focused on using *K8CreER^T2^* for postnatal lamin‐B1 deletion in scRNA‐seq. Due to the expression of both K5 and K8 in bipotent TEC progenitors, *K8CreER^T2^* should delete *Lmnb1*in all cTECs and a subset of mTECs.

**Figure 6:**
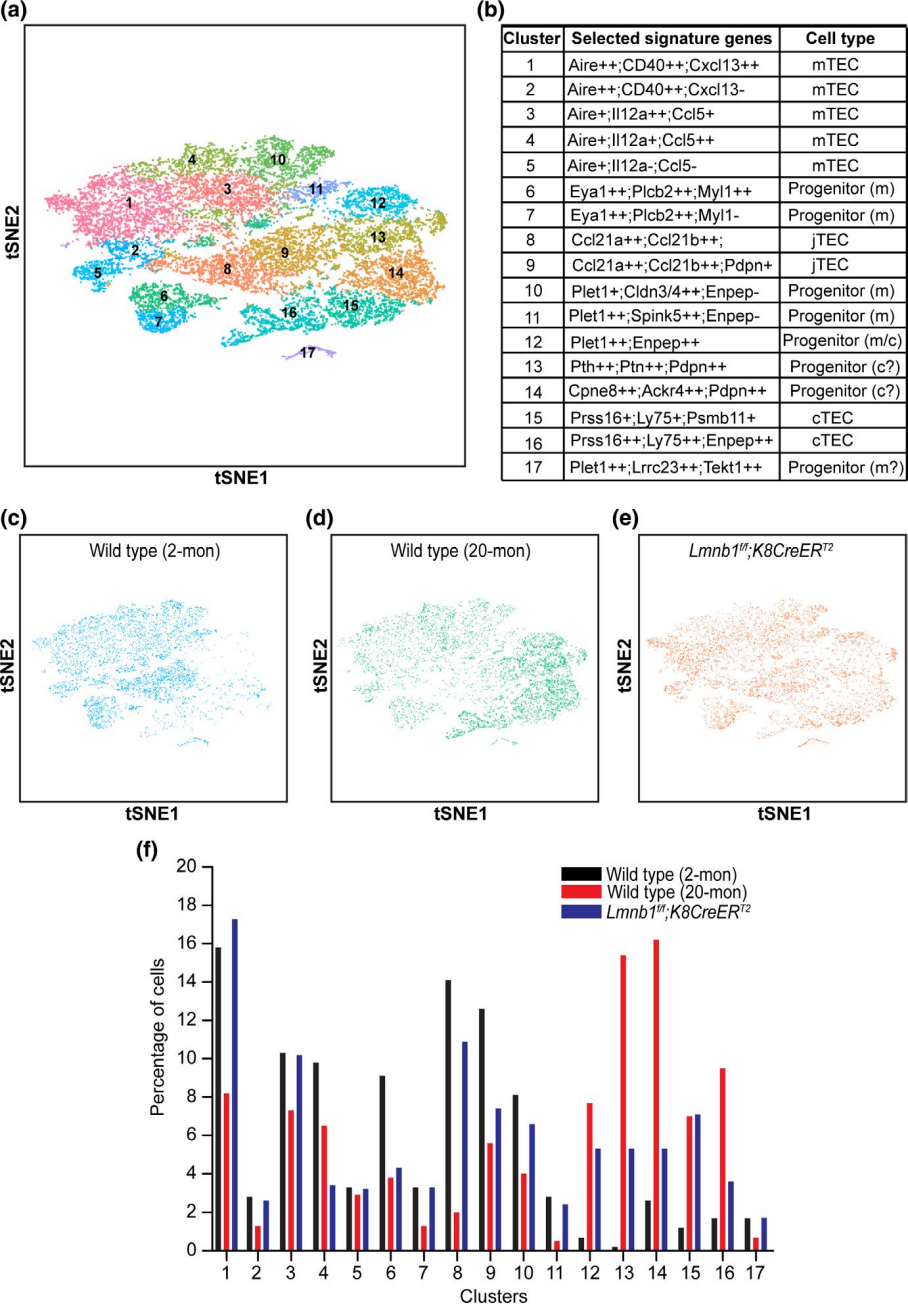
Single‐cell RNA‐seq of TECs in young, old, and postnatal lamin‐B1‐depleted thymus. (a) t‐Distributed Stochastic Neighbor Embedding (tSNE) plot of combined TECs from WT young (2‐mon; 5,872 cells), WT old (20‐mon; 6,973 cells), and TAM‐treated *Lmnb1^f/f^;K8CreER^T2^* (7,314 cells) mouse thymuses. Cell clusters are assigned to specific subgroups (1–17) based on differentially expressed marker genes. Each point is a single cell colored by cluster assignment. (b) List of selected marker genes and predicted subpopulations of individual TEC clusters. +: 2 ≤ fold change <3; ++: fold change ≥3; −: not enriched. (c–e) tSNE plots of TECs derived from WT young (c), WT old (d), and TAM‐treated *Lmnb1^f/f^;K8CreER^T2^*(e) mouse thymuses. Each point is a single cell colored by cluster assignment. (f) Percentage of the 17 TEC clusters shown in b in WT young, WT old, and TAM‐treated *Lmnb1^f/f^;K8CreER^T2^* mice

After quality control, we retained 5,872 cells with 2,149 median genes per cell from the young, 6,973 cells with 1,948 median genes per cell from the old, and 7,314 cells with 2,416 median genes per cell from the TAM‐treated *Lmnb1^f/f^;K8CreER^T2^* thymus. We combined all three samples to assess TEC heterogeneity. This also allowed us to assess how aging and postnatal lamin‐B1 depletion affect TEC composition. Unsupervised clustering analysis by t‐distributed stochastic neighborhood embedding (tSNE) identified 17 major clusters (C1‐17) of TECs in our dataset (Figure 6a,b; Supporting Information Table S3 for the full list). We found that the 17 clusters are present in all three thymus samples (Figure 6c–e), suggesting that our profiling identified the major TEC subsets in adult mouse thymus.

Based on the enriched mTEC signature genes such as Aire (Anderson et al., 2002), the cell clusters C1‐5 represent five differentiated mTEC subsets (Figure 6a,b). The cell clusters C6 and C7 do not appear to express the well‐characterized mTEC signature genes. However, when analyzing the available transcriptome profiles of the mouse neonatal cTECs and mTECs (St‐Pierre et al., 2013), we found that C6 and C7 express high levels of genes, such as Plcb2 and Myl1, that are specifically expressed in mTECs but not in cTECs in the neonatal mouse thymus. More importantly, these two clusters are enriched in expression of Eya1, a gene specifically expressed in a subset of mTEC progenitors identified by a recent scRNA‐seq of embryonic and neonatal thymus (Kernfeld et al., 2018). Thus, C6 and C7 may represent two previously unappreciated mTEC progenitor subsets in adult mouse thymus (Figure 6b).

We also identified several cell clusters, C8‐C11, that express different genes previously reported to be expressed in mTEC progenitors. Ccl21a and Ccl21b have been reported as markers for junctional TEC precursor (jTEC) that could give rise to fully differentiated mTECs (Miragaia et al., 2018; St‐Pierre et al., 2013). We found the C8 and C9 clusters express these two jTEC markers, but they can be further separated from one another based on differential expression of other genes such as Pdpn (Figure 6b), suggesting that the previously identified jTECs can be divided into two subsets. The C10 and C11 clusters represent two additional subsets of mTEC‐restricted progenitors because they express TEC progenitor‐specific marker Plet1 but lack the expression of Enpep, which encodes the antigen epitope for Ly51 (Figure 6b), one of the markers for bipotent TEC progenitor (Depreter et al., 2008; Ulyanchenko et al., 2016). By contrast, the cluster C12 expresses both Plet1 and Enpep and it corresponds to the newly identified Plet1^+^Ly51^+^ bipotent TEC progenitor that can effectively generate both cTECs and mTECs in the adult mouse thymus (Ulyanchenko et al., 2016). Finally, a minor cluster C17 may belong to the mTEC‐restricted progenitors because these cells express Plet1 and are enriched for genes specifically expressed in mTECs, such as Lrrc23 and Tekt1 (St‐Pierre et al., 2013).

The scRNA‐seq also revealed two different adult cTEC subsets as cluster C15 and C16 because they share genes previously identified for cTECs and they can be separated by some previously unappreciated differentially expressed genes (Figure 6b). Interestingly, we identified two clusters C13 and C14 that do not express previously defined mature cTEC signature genes, such as Prss16 and Ly75. However, their expression of specific genes enriched in cTECs but not in mTECs in neonatal thymus, including Pth, Ptn, Cpne8, and Ackr4 (St‐Pierre et al., 2013), suggests that they represent two new cTEC‐restricted short‐term progenitors. Together, our scRNA‐seq profiling has revealed new TEC progenitors and mature mTEC and cTEC subsets in adult thymus.

The identification of different TECs provides us the opportunity to assess how natural aging affects TEC composition changes and whether lamin‐B1 reduction in adult TEC contributes to aging‐associated TEC composition changes. We first examined the frequency of individual TEC subsets in WT young and old mouse thymuses. Consistent with the previous phenotypic analyses showing an overall reduction of mTECs upon aging (Chinn et al., 2012), we found that the percentages of most mTEC subsets (C1‐4) were significantly reduced upon aging (Figure 6f). Importantly, we identified a unique group of mTECs belonging to C5 that remained unchanged upon aging. By contrast, the two mature cTEC subsets (C15 and C16) exhibited a strong increase in percentage upon aging, which is consistent with the previous finding that total cTEC numbers increase with increased age (Chinn et al., 2012).

Of the progenitor cells identified, all the mTEC progenitor subsets (C6‐11) and the minor mTEC progenitor C17 exhibited a clear reduction upon aging (Figure 6f). Unexpectedly, we found that the bipotent TEC progenitor cluster C12 is present at a much higher frequency in the aged thymus than that of the young (Figure 6f). This suggests that the bipotent TECs can provide the source of thymic regeneration in aging mice, but their ability to differentiate into mature TECs may be compromised upon lamin‐B1 reduction.

When analyzing the impact of forced postnatal lamin‐B1 depletion by TAM treatment of the young *Lmnb1^f/f^;K8CreER^T2^* mice, we found an increase in all cTEC progenitor and mature cTEC subsets similar to those observed upon natural aging in WT controls (Figure 6d–f). Similar to the WT old thymuses, postnatal lamin‐B1 depletion in TAM‐treated *Lmnb1^f/f^;K8CreER^T2^* mice resulted in a reduction of C4 mTEC subset (Figure 6f). We also noticed a mild reduction of mTEC‐restricted TEC progenitors (C6, C8‐10) in TAM‐treated *Lmnb1^f/f^;K8CreER^T2^* thymus (Figure 6f). These indicate that *K8CreER^T2^* can delete *Lmnb1* alleles in some mTECs due to K8 expression in certain progenitor cells. Additionally, both the naturally aged WT thymus and the postnatal lamin‐B1‐depleted thymus exhibited a similar increase in the bipotent TEC progenitor subset C12 (Figure 6b and f). Taken together, scRNA‐seq analyses demonstrate that postnatal lamin‐B1 reduction contributes to the age‐associated changes of TEC composition, which can contribute to the disruption of thymic compartment and dysfunction.

### 2.8 Lamin‐B1 reduction in the adult TECs decreases thymopoiesis and accelerates age‐related loss of naïve T cells

Since the intact TEC compartments play essential roles in thymopoiesis, we next investigated the impact of lamin‐B1 reduction in the adult TECs on T‐cell development. We analyzed the CD4^+^SP and CD8^+^SP thymocytes and found a ~40%–45% reduction of these SP thymocytes in the *Lmnb1^f/f^;K8CreER^T2^*mice compared to the littermate controls one month after the last TAM injection (Supporting Information Figure [Link], [Link], [Link], [Link]a–c), indicating that lamin‐B1 plays an important role in cTECs to support efficient thymopoiesis in the adult mouse thymus. We also observed a similar but milder effect upon lamin‐B1 reduction in the adult mTECs in the *Lmnb1^f/f^;K5CreER^T2^*mice (data not shown), which is consistent with the less efficient *Lmnb1*deletion in these mice than that of the *Lmnb1^f/f^;K8CreER^T2^*mice. Thus, lamin‐B1 reduction upon aging can contribute to the gradual decline of the production of naïve T cells in the thymus.

Since the decline of naïve T cells in the peripheral immune system upon thymus involution contributes to the aging of the immune system (often referred to as immunosenescence) and reduced immune surveillance in elderly populations (Youm et al., 2012), we next assessed whether TAM‐induced lamin‐B1 deletion in the adult cTECs in thymus could affect the CD4^+^ and CD8^+^ T‐cell compartments in the peripheral immune organs such as the spleen and the mesenteric lymph node (mLN). Although no changes occurred in the frequency and the number of CD4^+^ and CD8^+^ naïve T cells (CD62L^+^CD44*^−^*) in these two immune organs by one month after the last TAM injection, by 6 months, we found a pronounced reduction of the naïve T cells in the *Lmnb1^f/f^;K8CreER^T2^*mice compared to their littermate controls (Supporting Information Figure [Link], [Link], [Link], [Link]d–i). This reduction was accompanied by a marked increase of CD4^+^ and CD8^+^ effector memory T cells (EM, CD62L*^−^*CD44^+^) and CD8^+^central memory T cells (CM, CD62L^+^CD44^+^) in these organs upon lamin‐B1 reduction (Supporting Information Figure [Link], [Link], [Link], [Link]d and g). Together, these findings demonstrate that lamin‐B1 reduction in adult TECs promotes thymic involution and causes changes in the peripheral T‐cell composition that are reminiscent of the age‐associated alterations in the peripheral immune system.

## 3 DISCUSSION

Since organ building and maintenance require structural proteins that support cell and tissue shapes, it is natural to assume that aging‐associated deterioration of these proteins would contribute to organ degeneration. The complex cellular composition in each organ, the difference in organ function, and the varying rates of organ aging have, however, made it challenging to identify structural proteins that have cell‐type‐specific functions in both building and maintaining organs. It is also difficult to define the causes and consequences of structural protein dysfunction in the same cell type later in life that contributes to organ degeneration. Our studies of the thymus demonstrate that among the three lamins found in mammals, lamin‐B1 is specifically required in TECs for thymus development and maintenance. Our findings further suggest that the age‐associated increase in intra‐thymic inflammation triggers senescence‐associated lamin‐B1 reduction in TECs, which promotes the gradual thymic degeneration and dysfunction.

Cellular senescence has been implicated in promoting organ aging (Campisi, 2013). However, senescence triggers multitudes of cellular changes, which make it difficult to tease apart the major and minor impact of these changes on organ aging. Additionally, although extensive studies of tissue culture cells have shown that many perturbations, including oxidative stress, DNA damage, oncogene transformation, and inflammation, can induce senescence in vitro (Ren et al., 2009), which cell type in a given organ is most sensitive to these senescence triggers and whether a specific senescence change of a given cell type makes a key contribution to organ aging remain unknown. By using mouse thymus known to experience age‐associated activation of inflammasomes (Youm et al., 2012), our studies provide a connection between a gradual increase of thymic inflammation to TEC senescence and lamin‐B1 reduction.

We show that both thymic macrophages and Sirpα^+^DCs exhibit age‐associated increase in IL‐1α, IL‐1β, IL‐6, and TNF‐α. Among these proinflammatory cytokines, we found that IL‐1β, IL‐6, and TNF‐α induced senescence and lamin‐B1 reduction in TECs in vitro. In addition, we observed that TEC lamin‐B1 was reduced upon acute induction of thymic involution by inflammatory challenges in vivo. These findings suggest that both chronic and acute thymic inflammation target TECs by inducing their senescence and lamin‐B1 reduction. Since lamin‐B1 reduction in TECs and the elevation of IL‐1α, IL‐1β, IL‐6, and TNF‐α in the thymic myeloid cells occur 2 months after birth, these thymic changes do not contribute to the first developmental phase of thymic involution but are correlated with the age‐associated thymic degeneration.

Previous studies have shown that *Foxn1*, whose expression is essential for TEC proliferation and differentiation, undergoes an age‐associated decline in TECs. Since Foxn1 deficiency in mouse TECs triggers premature thymic atrophy, the postnatal down‐regulation of Foxn1 has been generally accepted as a major mediator of age‐associated thymic involution (Chen et al., 2009; Cheng et al., 2010). More recently, however, it is recognized that mouse thymus displays a developmental and age‐associated phase of involution (Aw & Palmer, 2012; Shanley et al., 2009). The developmental involution occurs ~6 weeks after birth when thymus has produced a large number of naïve T cell required for establishing the peripheral immune system. Therefore, this initial thymic reduction could help to divert resources for other bodily needs without compromising the immune functions. On the other hand, the age‐associated involution is more gradual, and as thymic atrophy progresses, the immune system is negatively affected by the significant reduction of naïve T‐cell production. The differences in the two phases of thymic involution lead to the idea that they may be controlled by different mechanisms (Aw & Palmer, 2012; Shanley et al., 2009). Since the accumulation of Foxn1‐negative (Foxn1*^−^*) TECs reaches a plateau in ~50% of the total TECs within 10 weeks after birth (O'Neill et al., 2016; Rode et al., 2015), Foxn1 reduction soon after birth appears to be responsible for the developmental thymic reduction. Consistent with this, overexpression of *Foxn1* in TECs can delay, but not prevent the age‐dependent involution (Zook et al., 2011). Therefore, it is critical to identify additional TEC intrinsic factors that contribute to the age‐dependent phase of involution. Our studies suggest that intra‐thymic inflammation can lead to TEC senescence and lamin‐B1 reduction. Lamin‐B1 reduction in turn contributes to the age‐dependent thymic involution.

Using germline deletion of *Lmnb1* or *FN1Cre*‐mediated *Lmnb1* deletion in TECs during embryogenesis, we show that lamin‐B1 is required for the proper building of the cortical and medulla TEC compartments. This is consistent with a known function of B‐type lamins in tissue building. By carefully characterizing the roles of lamins in the thymus, we reveal a specific requirement of lamin‐B1 in TECs but not in the T‐cell lineage. Indeed, we found that lamin‐B1 helps to balance proper TEC differentiation from a common bipotent TEC progenitor and mediates terminal differentiation of cTECs as *Lmnb1*‐deficient thymus exhibits skewing toward the immature MHCII^low^cTEC dominant compartment.

To test whether lamin‐B1 reduction after birth could contribute to age‐associated thymic involution, we induced *Lmnb1* deletion in TECs starting from 2 months after birth and found it advanced thymic degeneration and dysfunction as judged by an increased disorganization of the TEC compartments, decreased naïve T‐cell production, and lymphopenia. By applying scRNA‐seq to TECs isolated from young WT, old WT, and postnatal lamin‐B1‐depleted thymuses, we identified 17 subsets of TEC progenitors and differentiated mTECs and cTECs in adult thymus. The signature genes we identified in different TEC subsets can be used as biomarkers for isolation and functional studies of specific TECs both in vitro and in vivo. The newly defined adult TEC subsets in this study have allowed us to explore in depth about how aging and postnatal lamin‐B1 depletion in TECs affect TEC composition changes. Importantly, we found an overall similar change of composition in TEC subsets in TAM‐treated *Lmnb1^f/f^;K8CreER^T2^*and WT aged mouse thymuses compared to that of the young. Specifically, we uncovered one mTEC progenitor subset that does not appear to change and two bipotent TEC progenitor subsets that increase upon aging or lamin‐B1 depletion. Together, these findings strongly suggest that lamin‐B1 reduction in TECs upon aging plays a key role in triggering the age‐associated alteration of TEC composition, thus contributing to thymic involution. The newly defined adult TEC subsets should also pave the way for further dissecting the functional contributions of individual TEC subsets to thymic aging and regeneration.

Additionally, we show that postnatal lamin‐B1 reduction contributes to significant transcriptome changes enriched for genes that function in cell proliferation, differentiation, cell–cell adhesion, and cytokine production. These gene expression changes help to explain why lamin‐B1 reduction upon aging or upon Cre‐mediated gene deletion in adult TECs causes TEC subtype composition change, thymic tissue disorganization, reduced naïve T‐cell production, and lymphopenia.

Lamins are known to influence the expression of genes found both in the lamina‐associated chromatin domains (LADs) and in the non‐LADs. Our recent studies of lamin null mouse embryonic stem cells (mESCs) have shown that lamins regulate the 3D chromatin organization by ensuring the proper condensation and position of LADs at the nuclear periphery (Zheng et al., 2018). The de‐condensation and detachment of LADs upon lamin loss can lead to altered gene expression by bringing the active chromatin domains into the close vicinity of inactive ones. Since lamin‐B reduction in *Drosophila* fat body cells and lamin‐B1 reduction in TECs both cause gene expression changes, it would be interesting to determine whether the changes are facilitated by 3D genome organization changes upon lamin‐B depletion.

## 4 EXPERIMENTAL PROCEDURES

### 4.1 Mice

All mouse experiments were approved by the Institutional Animal Care and Use Committee of Carnegie Institution for Science. *Lmnb1^f/f^* allele was derived from *Lmnb1* ^tm1a(EUCOMM)Wtsi^ by the EUCOMM project. The conditional *Lmnb2* allele, *Lmnb2^f/f^*, was derived from *Lmnb2* ^tm1a(KOMP)Wtsi^ (KOMP project) by breeding with ACTB‐FLPe mice to remove neomycin cassette flanked by Frt sites. *Lmna* ^f/f^ and *Lmnb1^−^* ^/^ *^−^* alleles were generated in house by Dr. Youngjo Kim and were reported previously (Kim & Zheng, 2013). All mice were further backcrossed to the C57BL/6 background for at least 6 generations. *LckCre*, *Foxn1Cre*, *K5CreET^T2^*, *K8CreER^T2^*, and *OT‐II* mice were purchased from the Jackson Laboratory. *K5CreET^T2^* and *K8CreER^T2^* mice were further backcrossed to the C57BL/6 background for at least 2 generations. 20‐mon‐old female C57BL/6 mice were purchased from National Institution on Aging. Other mice in cohort experiments at the indicated ages were raised by the mouse facility of Carnegie Institution for Science. All animals were housed under a 12‐/12‐hr light–dark cycle and fed ad libitum.

### 4.2 Primary TEC isolation and culture

Thymic epithelial cells were prepared according to a published protocol with modifications by using CD45 microbeads to enrich non‐T‐cell populations (Jain & Gray, 2014). In brief, thymic lobes were cut into 2‐mm pieces and then treated for 15 min at 37°C with an enzymatic mixture containing 0.25 U Liberase TM (Roche) and 0.1% DNase I (Sigma‐Aldrich) in RPMI 1640. The supernatant was collected, and the digestion was repeated twice. Cells were filtered through 100‐μm cell strainer and spun at 300 *g* for 5 min. The CD45^+^ cells were depleted by CD45 microbeads (Miltenyi Biotec 130‐052‐301), and the enriched CD45*^−^* cells were then stained with DAPI, CD45, EpCAM, Ly51, and I‐A/I‐E (BioLegend) and UEA‐1 (Vector Laboratories) for 20 min at 4°C followed by sorting with FACSAria^TM^ III (BD Biosciences). The TEC subpopulations that were DAPI negative were identified as mTEC: CD45*^−^*EpCAM^+^UEA‐1^+^Ly51*^−^*MHCII^+^; cTEC: CD45*^−^*EpCAM^+^UEA‐1*^−^*Ly51^+^MHCII^+^. FACS‐sorted TECs were further subjected to either downstream analyses or culture. For primary TEC culture, ~50,000 TECs were seeded in 48‐well culture plates and cultured for up to 7 days in Dulbecco's modified Eagle's medium nutrient F12 (Invitrogen) supplemented with 3 μg/ml insulin (Sigma‐Aldrich), 20 ng/ml epidermal growth factor (PeproTech), 100 unit/ml penicillin‐streptomycin, and 10% fetal bovine serum. Cultures were maintained at 37°C and 5% CO_2_, and the medium was changed every two days. For proinflammatory cytokine treatment, ~10,000 TECs were cultured in 96‐well culture plates with above‐mentioned medium supplemented with different concentrations of cytokines (10 ng/ml TNF‐α, PeproTech; 20 ng/ml IL‐1β, BioLegend; 20 ng/ml IL‐6, PeproTech; 20 ng/ml IL‐1α, BioLegend; 20 ng/ml G‐CSF; PeproTech). TECs were collected at indicated time points for RNA or protein extraction. For cell number count, ~50,000 TECs were seeded in 48‐well culture plates at day 0 and cultured primary TEC was collected after treating with indicated cytokines for 120 hr. TEC cells were then re‐suspended in 100 μl FACS buffer after trypsinization, and the cell numbers were recorded by running 30 μl cell solution in flow cytometry. The cell numbers were calculated by multiplying 3.3‐fold of the FACS recording.

### 4.3 Flow cytometry analysis of nuclear lamins

Single‐cell suspension of freshly dissected thymus was prepared as described for TEC isolation without depletion of CD45^+^ cells. Cells were first treated with TruStain fcX kit (BioLegend) to block CD16/32 and Zombie UV™ Fixable Viability Kit (Biolegend) to exclude dead cells before cell surface marker staining. The dead cells were identified as Zombie UV fixable viability dye‐positive populations. Surface marker staining was used to distinguish different cell types within the thymus: CD4/CD8 (T‐cell subsets); mTEC: CD45*^−^*EpCAM^+^UEA‐1^+^Ly51*^−^*; cTEC: CD45*^−^*EpCAM^+^UEA‐1*^−^*Ly51^+^. After surface marker labeling, staining of lamins follows the detailed protocol described in the True‐Nuclear Transcription Factor Kit (BioLegend). Alexa Fluor 647 antibody labeling kit (Life Technology) was used to prepare fluorochrome‐conjugated lamin antibody. In brief, 50 μg rabbit anti‐lamin‐B1 (Abcam), mouse anti‐lamin‐B2 (Invitrogen, E3), mouse anti‐lamin‐A/C (Active Motif), and control rabbit or mouse IgG (Santa Cruz) were labeled following the manufacturer's protocol. Fluorochrome‐conjugated antibodies were reconstituted at 0.5 mg/ml in PBS, and 0.1 μg of each antibody was used for staining 10^6^ cells in 100 μl volume. All samples were stained for 20 min at 4°C if not specifically indicated and then were immediately processed for FACS analyses by FACSAriaTM III (BD Biosciences). Data were analyzed with flowjo software (Tree Star).

### 4.4 Flow cytometry analysis and sorting

Single‐cell suspension of the thymus was prepared by passing minced tissue through a 40‐μm cell strainer. Cells were treated with TruStain fcX kit (BioLegend) to block CD16/32 before staining. All surface markers were stained for 20 min at 4°C, if not specifically indicated, and then were immediately processed for FACS analyses or sorting by FACSAriaTM III (BD Biosciences). The dead cells were identified as DAPI^+^ populations. The following FACS monoclonal antibodies were used for experiments: CD45, EpCAM, Ly51, I‐A/I‐E (MHCII), CD4, CD8, TCRβ, TCR γδ, CD69, TCRβV5.1, CD11b, F4/80, CD11c, and CD172 (Sirpα) (all from BioLegend) and UEA‐1 (Vector Laboratories). Macrophage and DC subsets were directly sorted into TRIzol (Life Technology) for RNA or 4X Laemmli sample buffer (Bio‐Rad) for protein extraction. Data were analyzed with FlowJo software (Tree Star).

### 4.5 Immunofluorescence staining

Freshly isolated thymuses were embedded in OCT (Leica Microsystems) and frozen immediately in −80°C. For keratin staining, 10‐μm sections were fixed in a 1:1 mixture of acetone and methanol at −20°C for 10 min and then washed with PBS for 3 times. After blocking, samples were stained with primary antibodies containing rat‐anti‐keratin 8 (Troma‐1, DSHB, 1:20) and rabbit anti‐keratin 5 (BioLegend, 1:300) at room temperature (RT) for 1 hr, followed by Alexa 488 goat anti‐rat and Alexa 568 goat anti‐rabbit (Invitrogen) secondary antibodies**.**Samples were then mounted with ProLong® Gold Antifade (Fisher Scientific) and allowed to dry overnight at RT before imaging. For lamin‐B1 immunostaining (IF) assay, cTECs and mTECs were collected by FACS sorting and then attached to glass slides with spike‐in RAW264.7 cells by cytospin (Shandon Cytospin 4) spun at 1,200 rpm for 5 min. After blocking, attached TECs were stained with primary antibodies containing rat‐anti‐keratin 8 (Troma‐1, DSHB, 1:20) and rabbit anti‐lamin‐B1 (1:500, Abcam), or rabbit anti‐keratin 5 (BioLegend, 1:300) and mouse anti‐lamin‐B1 (1:300, Santa Cruz) at room temperature (RT) for 1 hr, followed by Alexa 488 goat anti‐rat or anti‐rabbit (for keratins) and Alexa 568 goat anti‐rabbit or anti‐mouse (for lamin‐B1) secondary antibodies. Confocal images were acquired using a laser‐scanning confocal microscope (Leica SP5) with a 20× or 63× objectives. Images were processed using imagej.

### 4.6 RNA preparation and quantitative real‐time PCR

Thymic epithelial cells were isolated and enriched as described above. Total RNA was extracted following the manufacturer's protocol of Direct‐zol™ RNA MicroPrep Kit (Zymo Research R2060). Quantitative RT–PCR was performed using the iScript One‐Step RT‐PCR Kit (170–8,892; Bio‐Rad Laboratories) on a real‐time PCR detection system (CFX96; Bio‐Rad Laboratories). 50 ng of total RNA was reverse‐transcribed and amplified as follows: 50°C for 10 min, 95°C for 5 min, 95°C for 10 s, 60°C for 30 s, 72°C for 1 min. Steps 2–4 were repeated for 40 cycles. Each reaction was performed in triplicate, and the results of three independent experiments were used for statistical analysis. Relative mRNA expression levels were quantified using the ΔΔ*C* (*t*) method. Results were normalized to those for GAPDH, and primer sequences are listed as follows:

TNF‐α: forward primer (F), CTGTAGCCCACGTCGTAGC;

Reverse primer (R), TTGAGATCCATGCCGTTG.

IL‐1β: F, TGTAATGAAAGACGGCACACC; R, TCTTCTTTGGGTATTGCTTGG.

IL‐1α: F, CCGAGT TTCATTGCCTCTTT; R, ACTGTGGGAGTGGAGTGCTT.

IL‐6: F, CTCTGGGAAATCGTGGAAAT; R, CCAGTTTGGTAGCATCCATC.

P21: F, GACAAGAGGCCCAGTACTTC; R, GCTTGGAGTGATAGAAATCTGTC. P16: F, CGTACCCCG ATTCAGGTGAT; R, TTGAGCAGAAGAGCTGCTACGT. GAPDH: F, CGACTTCAACAGCAACTCCCACTCTTCC;

R, TGGGTGGTCCAGGGTTTCTTACTCCTT.

*Lmnb1*: F, AGTTTAGAGGGAGACTTGGAGG; R, TAAGGCTCTGACAGCGATTC

*Lmnb2*: F, TTAGGCCTCCAAAAGCAGG; R, CGTCATCTCCTGTTCCTTAGC

*Lmna*: F, TGAGAAGCGCACATTGGAG; R, GCGTCTGTAGCCTGTTCTC

### 4.7 Western blotting analysis

Whole‐cell lysates were generated using Laemmli sample buffer (Bio‐Rad) and diluted in SDS‐PAGE sample buffer. Cell lysates were separated in 10% or 15% SDS‐PAGE and then transferred onto nitrocellulose membranes. The membranes were blocked with 5% milk and probed with the following antibodies: rabbit anti‐lamin‐B1 (1:5,000; Abcam), mouse anti‐lamin‐A/C (1:5,000; Active Motif), rabbit anti‐TNF‐α (1:1,000; Cell Signaling), rabbit anti‐IL‐1β (1:1,000; Cell Signaling), rabbit anti‐IL‐6 (1:1,000; Novus biological), rabbit anti‐IL‐1α (1:2000, Santa Cruz), and mouse anti‐β‐actin (1:4,000; Sigma, AC‐15). Antibodies were detected with HRP‐conjugated anti‐mouse (1:10,000) or anti‐rabbit (1:10,000) antibodies and West Pico Substrate (Thermo Scientific).

### 4.8 Senescence‐associated (SA)‐β‐gal assay

Senescence β‐Galactosidase Staining Kit (Cell Signaling, 9860S) was used to detect β‐galactosidase activity at pH6.0. In brief, in vitro cultured TEC cells were fixed in the fixative solution for 10 min at RT. The fixed cells were washed twice with PBS and then incubated with β‐galactosidase staining solution containing X‐gal at 37°C overnight. After the blue color developed, bright‐field cell images were taken using an Axiovert 25 microscope (Carl Zeiss) connected to a Canon camera. Total 150 cells from three biological replicates were counted from each experimental group for quantification.

### 4.9 Tamoxifen (TAM)‐induced *Lmnb1* deletion

Stock solution of TAM (20 mg/ml; Sigma) was prepared by dissolving TAM powder in one volume of 100% ethanol at 55°C for 2 min and then mixed well with 9 volumes of prewarmed (55°C) corn oil. To induce *Lmnb1* (exon 2) deletion, 2‐mon‐old mice were treated with a single intraperitoneal (IP) injection of TAM (1 mg/10 g body weight/day) for five successive days and with 25 μg/ml TAM‐containing drinking water for the 1st month (Cheng et al., 2010). Thymus phenotypes were analyzed 1 month after the last TAM injection. Peripheral T‐cell phenotypes were analyzed 1 month or 6 months after the last TAM injection.

### 4.10 Endotoxin‐induced thymic involution model

The procedure follows a detailed protocol described in Billard et al. (2011). In brief, *Escherichia coli*‐derived lipopolysaccharide (LPS, Sigma‐Aldrich L‐2880) was reconstituted at 1 mg/ml in PBS. 2‐mon‐old female C57BL/6 mice were injected once intraperitoneally with LPS (100 µg) or PBS to induce thymic involution. Thymuses were collected at indicated time points for phenotype analyses and lamin measurement.

### 4.11 Single‐cell RNA‐sequencing and data processing

Immediately postsorting, CD45*^−^* DAPI*^−^* EpCAM^+^total TECs were run on the 10× Chromium and then single‐cell RNA‐seq libraries were generated using the Chromium Single Cell 3′ Reagent Kit (10× Genomics) by the core facility at the Embryology Department of Carnegie Institute for Science. Briefly, TEC single‐cell suspension (~1,000 cells per 1 µl PBS) was mixed thoroughly with Single Cell 3′ gel beads and partitioning oil into a Single Cell 3′ Chip (10× genomics) following the recommended protocol for the Chromium Single Cell 3′ Reagent Kit (v2 Chemistry). RNA transcripts of single cells were uniquely barcoded and reverse‐transcribed within the individual droplets. cDNA molecules were then pre‐amplified and pooled together followed by the final library construction. Libraries were sequenced by paired‐end 150‐bp reads on Illumina NextSeq 500. Postprocessing and quality control were performed by the same genomics core facility using the 10× Cell Ranger package (V2.1.1, 10× Genomics) as described by Zheng et al. (2017). Reads were aligned to mm10 reference assembly (v1.2.0, 10× Genomics). After cell demultiplexing and read counting, all three samples were combined using the cellranger aggr pipeline (Zheng et al., 2017). Cell visualization was done using the Loupe Cell Browser (10× Genomics).

### 4.12 Whole‐transcriptome shotgun sequencing (RNA‐seq)

By 1 month after the last TAM injection, ~5,000 cTECs from *Lmnb1^+/+^;K8CreER^T2^* and *Lmnb1^f/f^;K8CreER^T2^* mouse thymuses were sorted directly into TRIzol by FACS sorting. Total RNA was extracted following the manufacturer's protocol of Direct‐zol™ RNA MicroPrep Kit (Zymo Research). Poly‐A‐selected mRNA was purified, and sequencing libraries were built using Illumina TruSeq RNA Sample Prep Kit V2 (Illumina). Libraries were sequenced by single‐end 50‐bp reads on Illumina HiSeq 2000.

### 4.13 Bioinformatics

For RNA‐seq, low‐quality reads (quality score <20) were trimmed and the trimmed reads shorter than 36 bp were then filtered out. The remaining reads were further mapped to mouse genome (UCSC, mm9) by Tophat2. The number of reads falling into each gene was then counted using the custom scripts. The differentially expressed genes were called by edgeR (fold change ≥2, FDR < 0.05, and *p* value <0.05). GO term analyses of the differentially expressed genes were performed by DAVID.

## CONFLICT OF INTEREST

None declared.

## AUTHOR CONTRIBUTIONS

Yixian Zheng and Sibiao Yue conceived the idea. Sibiao Yue performed all the experiments. Yixian Zheng and Sibiao Yue interpreted the data and wrote the paper. Xiaobin Zheng analyzed and interpreted all the RNA‐seq data and performed GO term analyses of transcriptionally differentially expression of genes.
