# Supplementary figures and images for: Cell‐type‐specific role of lamin‐B1 in thymus development and its inflammation‐driven reduction in thymus aging
Source: Aging Cell. 2019 Apr 9;18(4):e12952. doi: 10.1111/acel.12952 (PMC6612680; doi:10.1111/acel.12952)

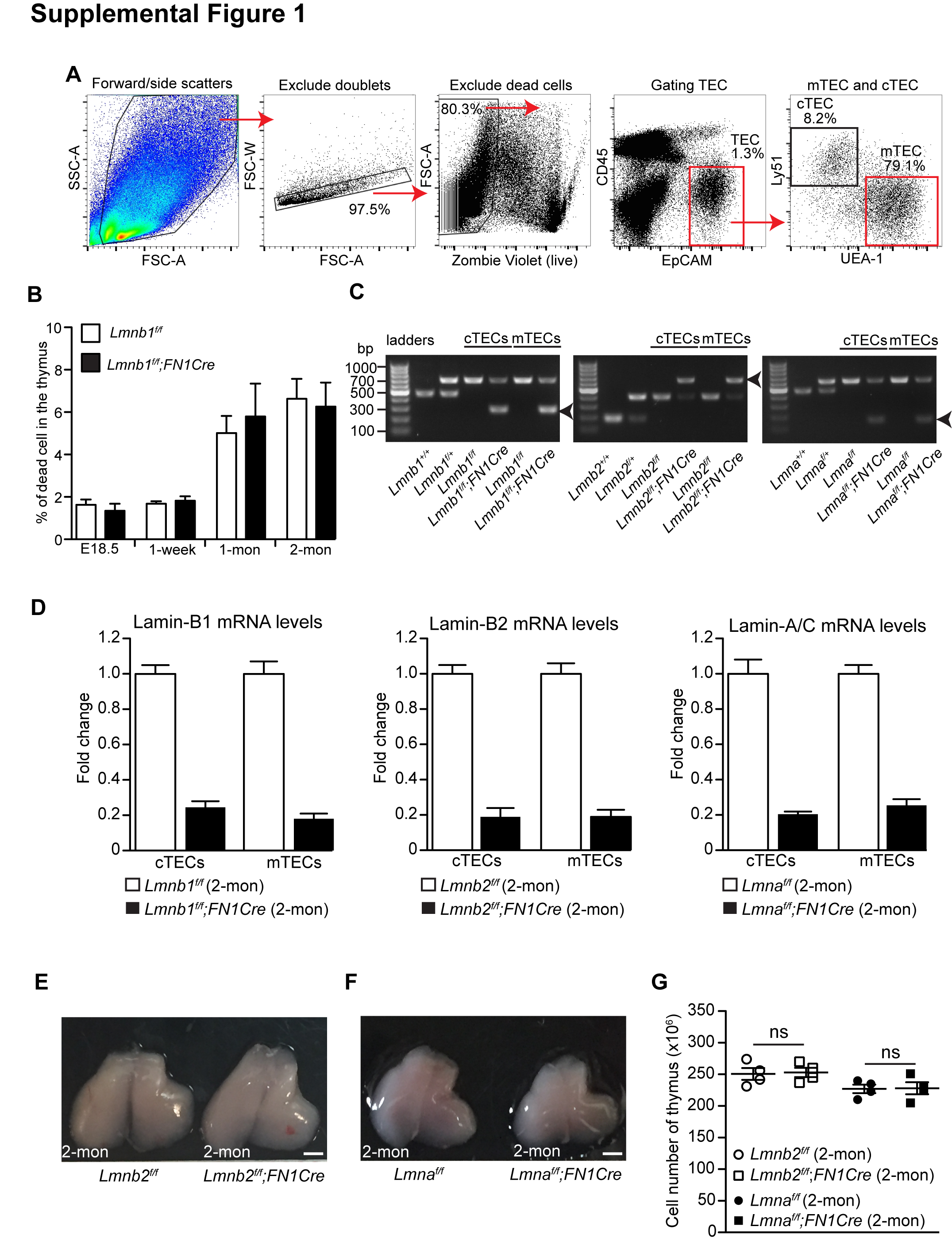

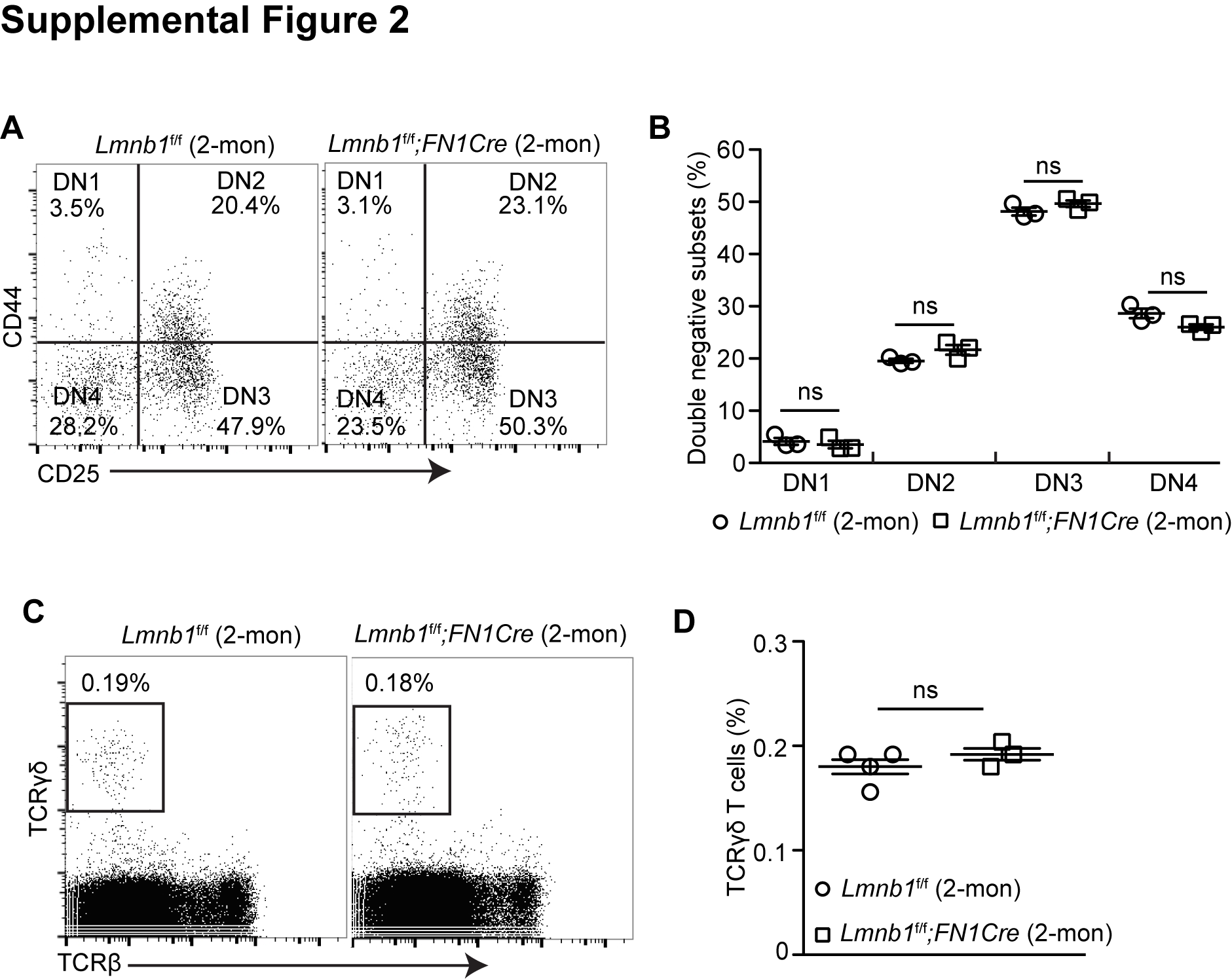


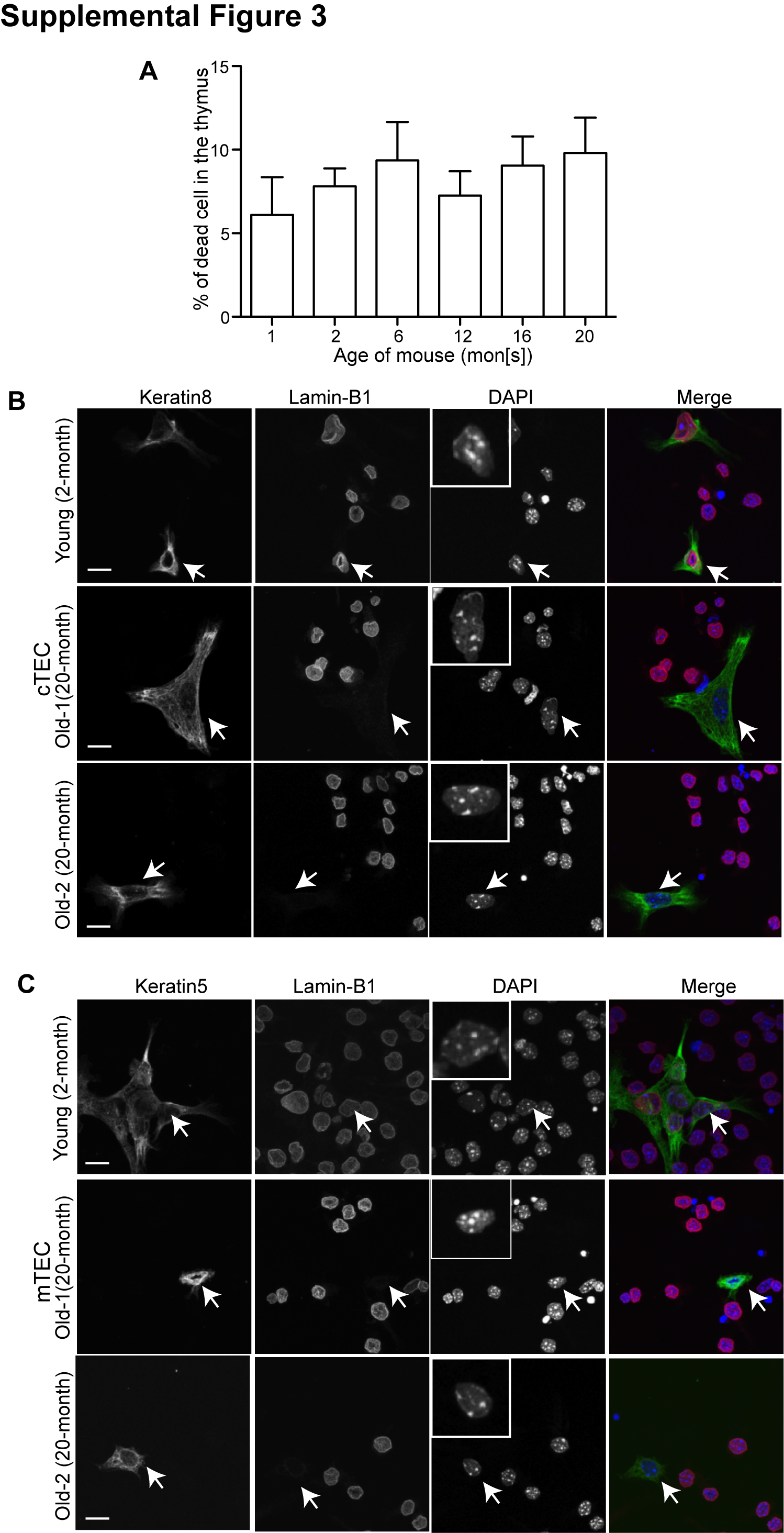


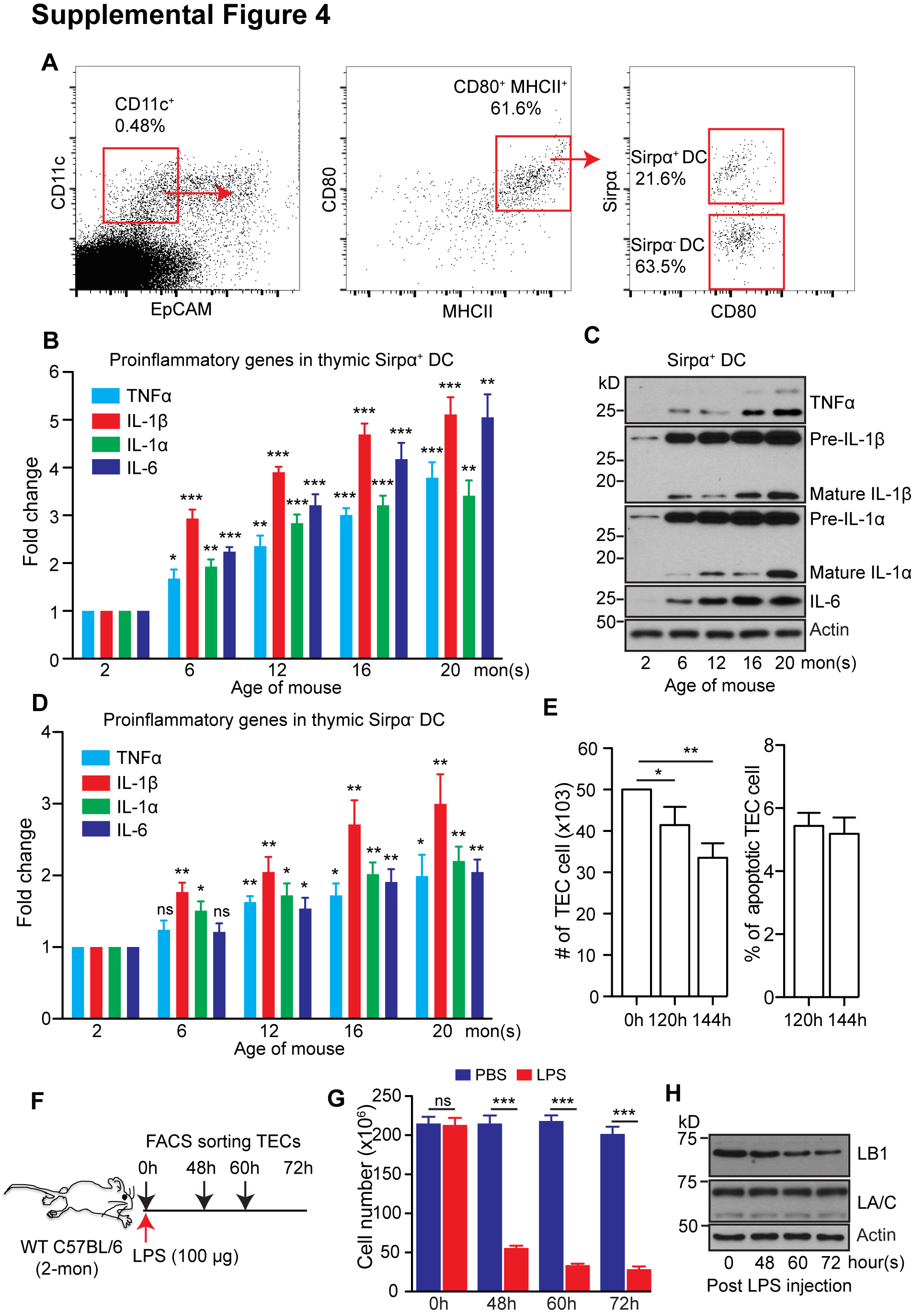


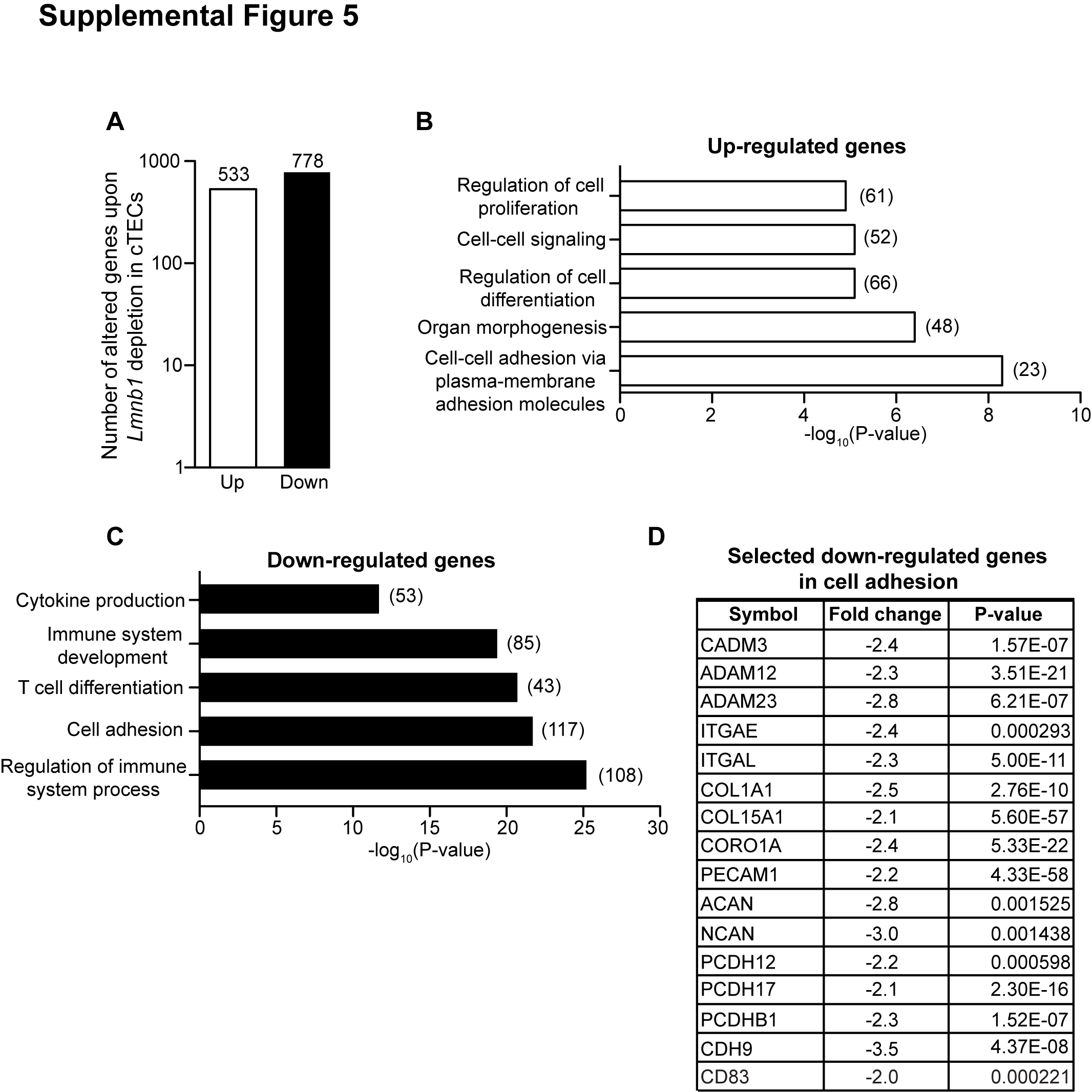


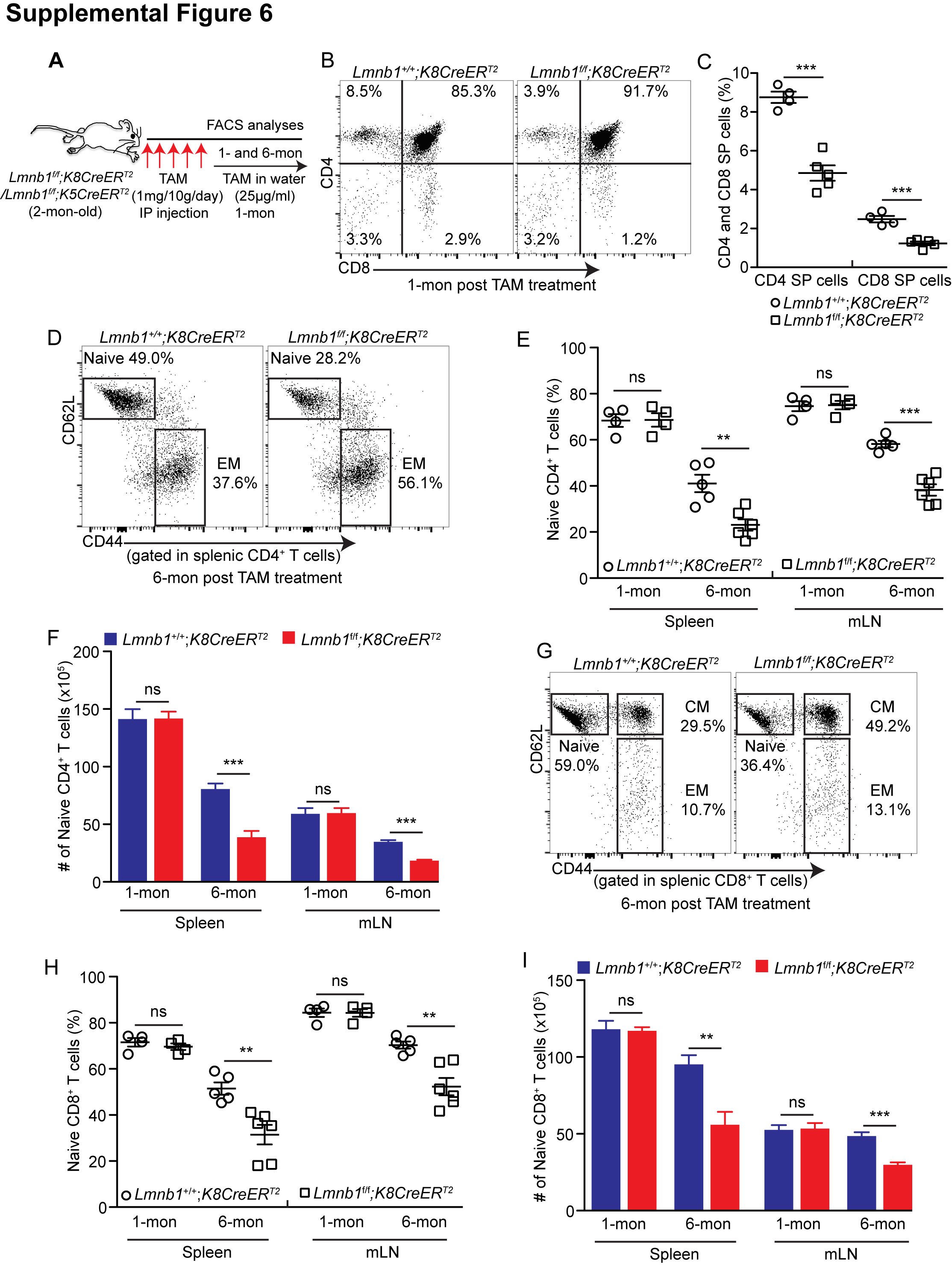

Supplement: Supplementary file 1 [file ACEL-18-e12952-s001.docx]
